# Prediction model based on MRI morphological features for distinguishing benign and malignant thyroid nodules

**DOI:** 10.1186/s12885-024-11995-3

**Published:** 2024-02-23

**Authors:** Tingting Zheng, Lanyun Wang, Hao Wang, Lang Tang, Xiaoli Xie, Qingyin Fu, Pu-Yeh Wu, Bin Song

**Affiliations:** 1 Department of Radiology, Minhang Hospital, Fudan University, No 170, Xinsong Road, Minhang District, 201199 Shanghai, China; 2 Department of Ultrasound, Minhang Hospital, Fudan University, No 170, Xinsong Road, Minhang District, 201199 Shanghai, China; 3 Department of Pathology, Minhang Hospital, Fudan University, No 170, Xinsong Road, Minhang District, 201199 Shanghai, China; 4 GE Healthcare, MR Research China, Beijing, China

**Keywords:** Thyroid nodule, Magnetic resonance imaging, Prediction model, Benign, Malignant

## Abstract

**Background:**

The low specificity of Thyroid Imaging Reporting and Data System (TI-RADS) for preoperative benign-malignant diagnosis leads to a large number of unnecessary biopsies. This study developed and validated a predictive model based on MRI morphological features to improve the specificity.

**Methods:**

A retrospective analysis was conducted on 825 thyroid nodules pathologically confirmed postoperatively. Univariate and multivariate logistic regression were used to obtain β coefficients, construct predictive models and nomogram incorporating MRI morphological features in the training cohort, and validated in the validation cohort. The discrimination, calibration, and decision curve analysis of the nomogram were performed. The diagnosis efficacy, area under the curve (AUC) and net reclassification index (NRI) were calculated and compared with TI-RADS.

**Results:**

572 thyroid nodules were included (training cohort: *n* = 397, validation cohort: *n* = 175). Age, low signal intensity on T2WI, restricted diffusion, reversed halo sign in delay phase, cystic degeneration and wash-out pattern were independent predictors of malignancy. The nomogram demonstrated good discrimination and calibration both in the training cohort (AUC = 0.972) and the validation cohort (AUC = 0.968). The accuracy, sensitivity, specificity, PPV, NPV and AUC of MRI-based prediction were 94.4%, 96.0%, 93.4%, 89.9%, 96.5% and 0.947, respectively. The MRI-based prediction model exhibited enhanced accuracy (NRI>0) in comparison to TI-RADSs.

**Conclusions:**

The prediction model for diagnosis of benign and malignant thyroid nodules demonstrated a more notable diagnostic efficacy than TI-RADS. Compared with the TI-RADSs, predictive model had better specificity along with a high sensitivity and can reduce overdiagnosis and unnecessary biopsies.

**Supplementary Information:**

The online version contains supplementary material available at 10.1186/s12885-024-11995-3.

## Key points

1. MRI morphological features were predictors of malignant thyroid nodules.
2. Prediction model achieved a high AUC, sensitivity, and specificity in predicting malignant thyroid nodules.
3. Prediction mode outperformed TI-RADSs in diagnosing malignant thyroid nodules.

**Supplementary Information:**

The online version contains supplementary material available at 10.1186/s12885-024-11995-3.

## Introduction

Thyroid nodules are very common, and with the advances of high-resolution ultrasound, the detection rate in the general population ranges from 19 to 68%. The majority of thyroid nodules are benign, and only a small fraction is clinically significant [1–3]. The incidence of thyroid cancer continues to rise and is ranked as the fifth most common cancer among women in the United States currently [4]. The clinical management of thyroid nodules depends on the benignity or malignancy [5] and early qualitative diagnosis plays a crucial role in optimizing treatment and improving patient outcomes.

Various Ultrasonography risk stratification systems have been developed to effectively manage of thyroid nodules, including the American College of Radiology Thyroid Imaging Reporting and Data System (ACR- TIRADS) [6], Korean TIRADS (K-TIRADS) [7], European TIRADS (EU-TIRADS) [8], Kwak-TIRADS [9] and Chinese-TIRADS (C-TIRADS) [10]. While these TI-RADSs demonstrated high sensitivity (>90%) in identifying nodules, their specificity remains relatively unsatisfactory [11]. Additionally, differentiated thyroid cancer accounts for over 90% of cases, and has an excellent prognosis, with a 5-year survival rate exceeding 98% [12]. The high sensitivity and relatively low specificity of TI-RADS systems have led to the diagnosis of numerous thyroid nodules that lack clinical significance, resulting in unnecessary fine-needle aspiration (FNA) and overtreatment. FNA is considered the gold standard for the preoperative diagnosis of thyroid cancer. However, it is an invasive procedure, and approximately 20–30% of the puncture results are either nondiagnostic or of uncertain significance [13, 14].

Magnetic resonance imaging (MRI) has gained significant popularity in the diagnosis of head and neck tumors due to its numerous advantages, including multi-parameter measurement, arbitrary planar imaging, low risk of contrast allergy, no ionizing radiation, and high soft tissue contrast [15]. In recent years, there has been a growing trend in utilizing MRI for the preoperative evaluation of thyroid nodules [16–20]. However, few studies have explored the use of morphological features on multiparametric MRI to assess the benignity or malignancy of thyroid nodules.

In this study, we investigated the value of MRI morphological features in distinguishing between benign and malignant thyroid nodules. We also developed and validated a prediction model and compared its performance with the ultrasound-based TI-RADS system.

## Materials and methods

### Patients and study design

This study was a retrospective observational study conducted in accordance with the Declaration of Helsinki. Informed consent requirements were waived due to the retrospective nature of the study by the Institutional Review Board of Fudan University Minhang Hospital (2020-008-01 K).

Consecutive patients who underwent surgical thyroidectomy at our institution from January 2017 to December 2022 were retrospectively analyzed. Inclusion criteria were as follows: (1) patients underwent preoperative thyroid MRI; (2) nodules with postoperative pathological confirmation as either benign or malignant. Exclusion criteria were as follows: (1) diffuse bilateral lesions of different pathological types; (2) poor image quality with severe artefacts that cannot be used for diagnostic analysis; (3) patients who underwent FNA or partial thyroidectomy prior to MRI; (4) unclear postoperative pathological findings; (5) absence of nodules on MRI; (6) incomplete imaging data; and (7) lesions<5 mm. The surgical indications for thyroid nodules include those categorized as TI-RADS grade≥4, indicating a high suspicion of thyroid cancer, as well as symptomatic benign thyroid tumors resulting from compression, hyper-functioning thyroid adenomas, or concomitant hyperthyroidism.

### MRI Acquisition

MRI examinations were performed on a GE Healthcare 1.5T MRI scanner (Excite HD; GE Healthcare, Milwaukee, WI, USA) with an 8-channel phased-array thyroid coil (Shanghai Chenguang Medical Technologies, Shanghai, China).

The MRI protocols included: (1) coronal fat-suppressed T2WI; (2) axial T1-weighted imaging (T1WI); (3) axial fat-suppressed T2W; (4) diffusion weighted imaging (DWI), b value = 0 and 800 s/mm^2^; (5) multiphasic contrast-enhanced T1WI (MCE-T1WI). Contrast agent (Magen Vixen; Bayer Pharmaceuticals, Berlin, Germany) was injected at a dose of 0.2 ml/kg and rate of 3 ml/s, followed by 15 ml saline flush. Six sequential MCE-T1WI scans were performed at 30 s, 60 s, 120 s, 180 s, 240 s, and 300 s after the contrast agent injection. Patients were instructed to hold their breath during the scan. The total scan duration was approximately 16 min. Table S1 lists the detailed MRI acquisition parameters.

### MRI morphological analysis

Two radiologists, each with 5 and 9 years of experience in diagnostic thyroid MRI, independently evaluated the MRI images using the Advantage Workstation 4.5 workstation (GE Healthcare, Waukesha, WI, USA) and Picture Archiving and Communication System (PACS). Both radiologists were unaware of the pathological results of lesions and consensus was reached in cases where there was a disagreement.

The following parameters were utilized to assess the lesion: (1) size of the lesion, measured by the diameter of the largest dimension of the nodule, classified as 5-10 mm, 10-40 mm or ≥4 cm); (2) number of nodules, classified as unifocal or multifocal. The qualitative MRI morphological features are as follows: (1) non-enhanced features, including light pearl sign, black-white flower sign, restricted diffusion, cystic degeneration, flow-void signal, high signal intensity on T2WI, high signal intensity on T1WI, and low signal intensity on T2WI; (2) contrast-enhanced features, including enhancement patterns like no enhancement, gap-filling enhancement, pseudocapsule, hyperintense on T2WI with enhancement, wash-out pattern, fissure-filling enhancement, reversed halo sign in delay phase, hyperenhancement in early phase and change of lesion size in multiphasic enhancement. Detailed definitions and illustrations of the MRI morphological features are shown in **Appendix S1**.

### TI-RADS

Two experienced US specialists with over 10 years of experience, who were unaware of the histopathological findings, performed the retrospective analysis of the US feature of thyroid nodules and reached a consensus. The US features evaluated encompassed composition, echogenicity, margins, shape, and calcification. All thyroid nodules were then classified according to the ACR-TIRADS, K-TIRADS, EU-TIRADS, Kwak-TIRADS, and C-TIRADS. In the cases of ACR-TIRADS, K-TIRADS, and EU-TIRADS, nodules categorized as ≥4 or 5 were considered to be malignant. For Kwak-TIRADS and C-TIRADS, nodules categorized as ≥4b and 4c, respectively, were regarded as malignant. The diagnostic performance of these five different TIRADS systems was subsequently calculated.

### Nomogram construction and evaluation

Univariate and multivariate logistic stepwise regression analysis were employed to identify independent predictors in the training cohort and then a nomogram was developed. The optimal model was selected based on the Akaike Information Criteria.The goodness-of-fit of the model was assessed using the Hosmer-Lemeshow test, with a significance level of *P*≥0.05 indicating a good fit. To evaluate the performance of the nomogram, receiver operating characteristic (ROC) analysis, calibration curve analysis, and decision curve analysis (DCAs) were conducted.

### Construction and performance of the MRI-based prediction model

The risk score derived from the regression coefficients (β coefficients) of the independent predictors by multiples of the minimum β coefficient (multiples are rounded to the nearest whole number) was used to develop a risk scoring system (RSS). To establish an optimal cut-off value for the risk score, the Yorden-index is maximized. The MRI-based prediction model was constructed using the aforementioned RSS and specific morphological features. The diagnostic performance of the model was evaluated by assessing sensitivity, specificity, accuracy, positive predictive value (PPV), negative predictive value (NPV) and area under the ROC curve (AUC), and compared it with five different US TI-RADS. The AUCs were compared using the Delong test. The Net reclassification index (NRI) was utilized to determine the enhancement in predictive accuracy of the model.

### Statistical analysis

All statistical tests were carried out using SPSS statistical software 26.0, R software 4.2.0 (http://www.r-project.org, and Medcalc Software (version 20.100). Continuous variables were represented as mean±standard deviation (SD), while categorical variables were expressed as percentages. The t-test was employed to compare continuous variables, whereas the chi-square test or Fisher’s exact test was used to compared categorical variables. Concordance between two radiologists was assessed using the Kappa concordance test. The construction of the nomogram was accomplished using the R software package “rms”. Statistical analyses were conducted with two-tailed p values and 95% confidence intervals (CI). A significance level of *P*<0.05 was deemed statistically significant.

## Results

### Clinicopathological characteristics

**Fig. 1:**
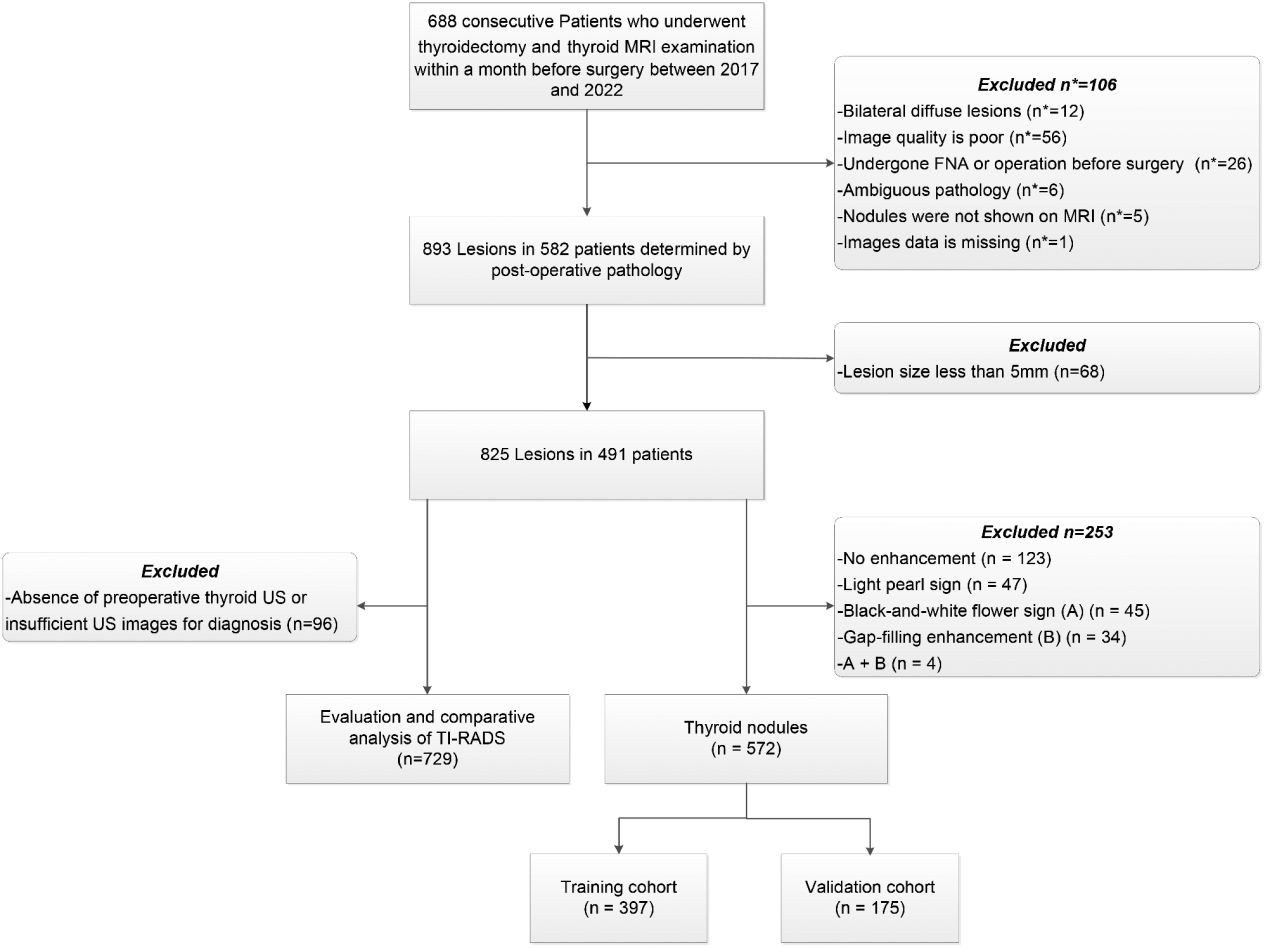
Flowchart of the study inclusion process

The flowchart illustrating the inclusion process is shown in Fig. 1. A total of 825 lesions from 491 patients were finally included in the study, comprising 508 benign and 317 malignant lesions. We removed lesions with specific features that were not included in the modelling below. There were 572 lesions remaining (337 benign and 235 malignant) finally. These lesions were randomly divided into the training cohort (397 thyroid nodules, including 230 benign and 167 malignant nodules) and the validation cohort (175 thyroid nodules, 107 benign and 68 malignant nodules) at a 7:3 ratio. The clinicalcopatholgical characteristics of 572 thyroid nodules are shown in Table 1. The pathological types of all thyroid nodules are shown in Table S2.

**Table 1:** Patient characteristics

Variable	Training cohort (*n* = 397)			Validation cohort (*n* = 175)			*p* value
	Benign	Malignant	*p* value	Benign	Malignant	*p* value	
Age, mean ± SD, years	53.49 ± 12.78	45.99 ± 14.63	<0.001*	53.59 ± 12.89	46.19 ± 14.15	<0.001*	0.764
Thyroid volume, cm^3^	22.70 ± 29.53	11.49 ± 15.37	<0.001*	20.58 ± 19.44	10.81 ± 13.55	<0.001*	0.569
Gender			0.083			0.773	0.842
Male	45 (19.6)	45 (26.9)		26 (24.3)	15 (22.1)		
Female	185 (80.4)	122 (73.1)		81 (75.7)	53 (77.9)		
Number			<0.001*			<0.001*	0.586
Unifocal	47 (20.4)	80 (47.9)		22 (20.6)	33 (48.5)		
Multifocal	183 (79.6)	87 (52.1)		85 (79.4 )	35 (51.5)		
Location			0.278			0.074	0.269
Left lobe	108 (47.0)	65 (38.9)		60 (56.1)	27 (39.7)		
Right lobe	113 (49.1)	95 (56.9)		41 (38.3)	38 (55.9)		
Isthmus	9 (3.9)	7 (4.2)		6 (5.6)	27 (39.7)		
Size			<0.001*			<0.001*	0.387
0.5-1 cm	65 (28.3)	89 (53.3)		23 (21.5)	38 (55.9)		
1-4 cm	128 (55.7)	67 (40.1)		63 (58.9)	23 (33.8)		
≥4 cm	37 (16.1)	11 (6.6)		21 (19.6)	7 (10.3)		
Hashimoto’s thyroiditis			0.089			0.041*	0.007*
Absent	192 (83.5)	128 (76.6)		100 (93.5)	57 (83.8)		
Present	38 (16.5)	39 (23.4)		7 (6.5)	11 (16.2)		

Data are expressed as the number of nodules, with percentages in parentheses. Abbreviation: SD,Standard deviation. *, *P*<0.05

### MRI morphological features

The statistical results of MRI-based specific morphological features are provided in Table S3. Significantly, nodules exhibiting no enhancement (*n* = 123) and the presence of the light pearl sign (*n* = 47) were exclusively observed in benign cases. Conversely, the black-and-white flower sign (*n* = 49) and gap-filling enhancement (*n* = 38) were predominantly detected in malignant nodules. The morphological features of thyroid nodules are described in Table 2. There was no statistically significant difference identified in the morphological features between the training and validation cohorts (*P*>0.05) excepting hypointense on T2WI. Kappa coefficients for different features ranged from 0.725 to 0.92.

**Table 2:** MRI qualitative features of thyroid nodules in training and validation cohorts

Variables	Training cohort (*n* = 397)			Validation cohort (*n* = 175)			Kappa	p value
	Benign	Malignant	p	Benign	Malignant	p		
High signal intensity on T2WI			<0.001*			<0.001*	0.879	0.123
Absence	58 (25.2)	133 (79.6)		19 (17.8)	53 (77.9)			
Presence	172 (74.8)	34 (20.4)		88 (82.2)	15 (22.1)			
High signal intensity on T1WI			<0.001*			<0.001*	0.884	0.867
Absence	160 (69.6)	160 (95.8)		76 (71.0)	64 (94.1)			
Presence	70 (30.4)	7 (4.2)		31 (29.0)	4 (5.9)			
Hypointense on T2WI			<0.001*			<0.001*	0.725	0.015*
Absence	187 (81.3)	45 (26.9)		98 (91.6)	23 (33.8)			
Presence	43 (18.7)	122 (73.1)		9 (8.4)	45 (66.2)			
Restricted diffusion			<0.001*			<0.001*	0.914	0.312
Absence	211 (91.7)	23 (13.8)		100 (93.5)	11 (16.2)			
Presence	19 (8.3)	144 (86.2)		7 (6.5)	57 (83.8)			
Cystic degeneration			0.002*			0.034*	0.92	0.924
Absence	201 (87.4)	161 (96.4)		94 (87.9)	66 (97.1)			
Presence	29 (12.6)	6 (3.6)		13 (12.1)	2 (2.9)			
Flow-void signal			0.316			0.907	0.831	0.111
Absence	215 (93.5)	160 (95.8)		97 (90.7)	62 (91.2)			
Presence	15 (6.5)	7 (4.2)		10 (9.3)	6 (8.8)			
Reversed halo sign in delay phase			<0.001*			<0.001*	0.818	0.255
Absence	213 (92.6)	35 (21.0)		103 (96.3)	15 (22.1)			
Presence	17 (7.4)	132 (79.0)		4 (3.7)	53 (77.9)			
Pseudocapsule			<0.001*			<0.001*	0.899	0.151
Absence	109 (47.4)	154 (92.2)		44 (41.1)	61 (89.7)			
Presence	121 (52.6)	13 (7.8)		63 (58.9)	7 (10.3)			
Fissure-filling enhancement			0.001*			0.005*	0.874	0.082
Absence	195 (84.8)	161 (96.4)		84 (78.5)	64 (94.1)			
Presence	35 (15.2)	6 (3.6)		23 (21.5)	4 (5.9)			
Hyperintense on T2WI with enhancement			<0.001*			0.001*	0.893	0.676
Absence	117 (50.9)	142 (85.0)		55 (51.4)	56 (82.4)			
Presence	113 (49.1)	25 (15.0)		52 (48.6)	12 (17.6)			
Wash-out pattern			<0.001*			0.002*	0.909	0.325
Absence	142 (61.7)	49 (29.3)		66 (61.7)	26 (38.2)			
Presence	88 (38.3)	118 (70.7)		41 (38.3)	42 (61.8)			
Hyperenhancement in early phase			<0.001*			0.001*	0.900	0.399
Absence	169 (73.5)	156 (93.4)		76 (71.0)	62 (91.2)			
Presence	61 (26.5)	11 (6.6)		31 (29.0)	6 (8.8)			
Change of lesion size in multiphasic enhancement			<0.001*			<0.001*	0.743	0.239
Absence	117 (50.9)	17 (10.2)		58 (54.2)	10 (14.7)			
Presence	113 (49.1)	150 (89.8)		49 (45.8)	58 (85.3 )			

Data are expressed as the number of nodules, with percentages in parentheses

Abbreviation: OR,odds ratio;CI, confidence interval; T2WI, T2-weighted imaging; T1WI, T1-weighted imaging. *p< 0.05

Table 3 show the factors associated with malignant thyroid nodules. In the univariate and multivariate analysis, independent predictors of malignant thyroid nodules were age (OR=1.96, *P*<0.001), low signal intensity on T2WI (OR = 3.92, *P* = 0.003), diffusion restriction (OR = 39.54, *P* < 0.001), reversed halo sign in delay phase (OR = 19.26, *P* < 0.001), cystic degeneration (OR = 4.44, *P* = 0.031) and wash-out pattern (OR = 3.04, *P* = 0.009).

**Table 3:** Univariate and multivariate analysis to identify factors associated with malignant thyroid nodule and a scoring system developed from β coefficient in training cohort

Variables	Univariate analysis		Multivariate analysis		β coefficient	Risk Score
	Odds ratio (95% CI)	p value	Odds ratio (95% CI)	p value		
Gender (male, female)	1.52 (0.95-2.43)	0.084				
Age (≤48y, >48y)	3.07 (2.02-4.66)	<0.001*	1.96 (0.88-4.36)	0.101	0.672	2
Lesion number (unifocal, multifocal)	0.24 (0.15-0.37)	<0.001*				
Tumor size (<1 cm, 1-4 cm, ≥4 cm)	0.43 (0.31-0.60)	<0.001*				
High signal intensity on T2WI	0.09 (0.05-0.14)	<0.001*				
High signal intensity on T1WI	0.10 (0.04-0.22)	<0.001*				
Low signal intensity on T2WI	11.79 (7.32-18.98)	<0.001*	3.92 (1.57-9.78)	0.003*	1.367	4
Restricted diffusion	69.53 (36.56-132.24)	<0.001*	39.54 (16.30-95.89)	<0.001*	3.677	11
Reversed halo sign in delay phase	47.25 (25.44-87.79)	<0.001*	19.26 (7.53-49.25)	<0.001*	2.958	9
Pseudocapsule	0.08 (0.04-0.14)	<0.001*				
Fissure-filling enhancement	0.21 (0.09-0.51)	0.001*				
Cystic degeneration	0.26 (0.10-0.64)	0.003*	4.44 (1.15-17.12)	0.031*	1.490	4
Hyperintense on T2WI with enhancement	0.18 (0.11-0.30)	<0.001*				
Flow-void signal	0.63 (0.25-1.57)	0.320				
Wash-out pattern	3.89 (2.54-5.95)	<0.001*	3.04 (1.32-6.98)	0.009*	1.112	3
Hyperenhancement in early phase	0.20 (0.10-0.38)	<0.001*				
Change of lesion size in multiphasic enhancement	9.14 (5.20-16.07)	<0.001*				

Abbreviations: CI, confidence interval; T2WI, T2-weighted imaging; T1WI, T1-weighted imaging. *P<0.05

### TI-RADS

A total of 729 nodules were included in the final analysis. Out of these, 452 nodules (62.0%) were determined to be benign, while 277 nodules (38.0%) were classified as malignant. Table S4 presents the distribution of TI-RADS classification. The malignancy rate exhibited a progressive increase with higher grade classifications, and the differences between the classifications were found to be statistically significant (*P*<0.001).

### Development and validation of the nomogram

**Fig. 2:**
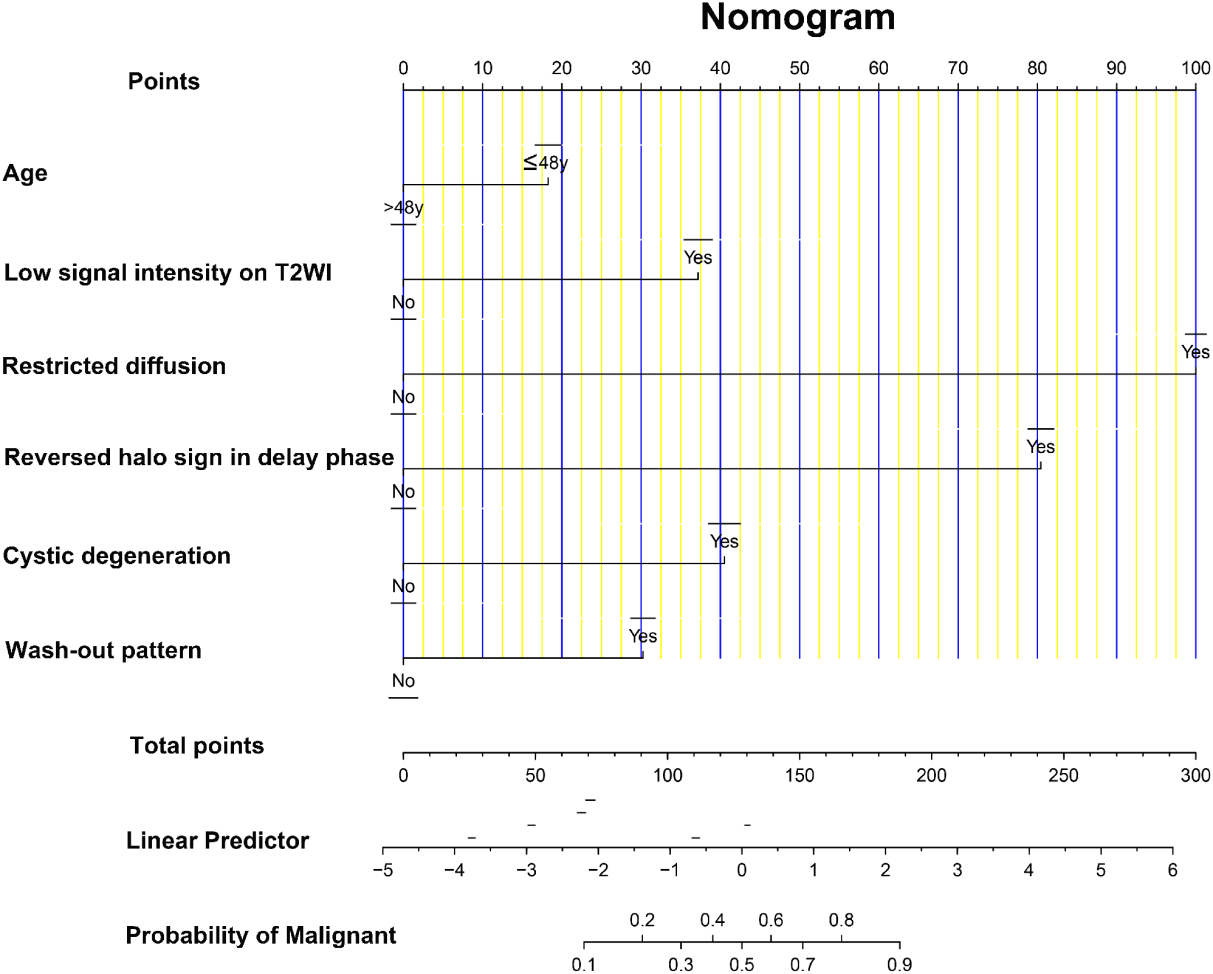
Nomogram for preoperative estimation of malignant thyroid nodule. When using the nomogram, find the position of each feature on the axis, and identify the corresponding point vertically. On the bottom scale, the points of all features were added up and converted into the probability of a malignant thyroid nodule

**Fig. 3:**
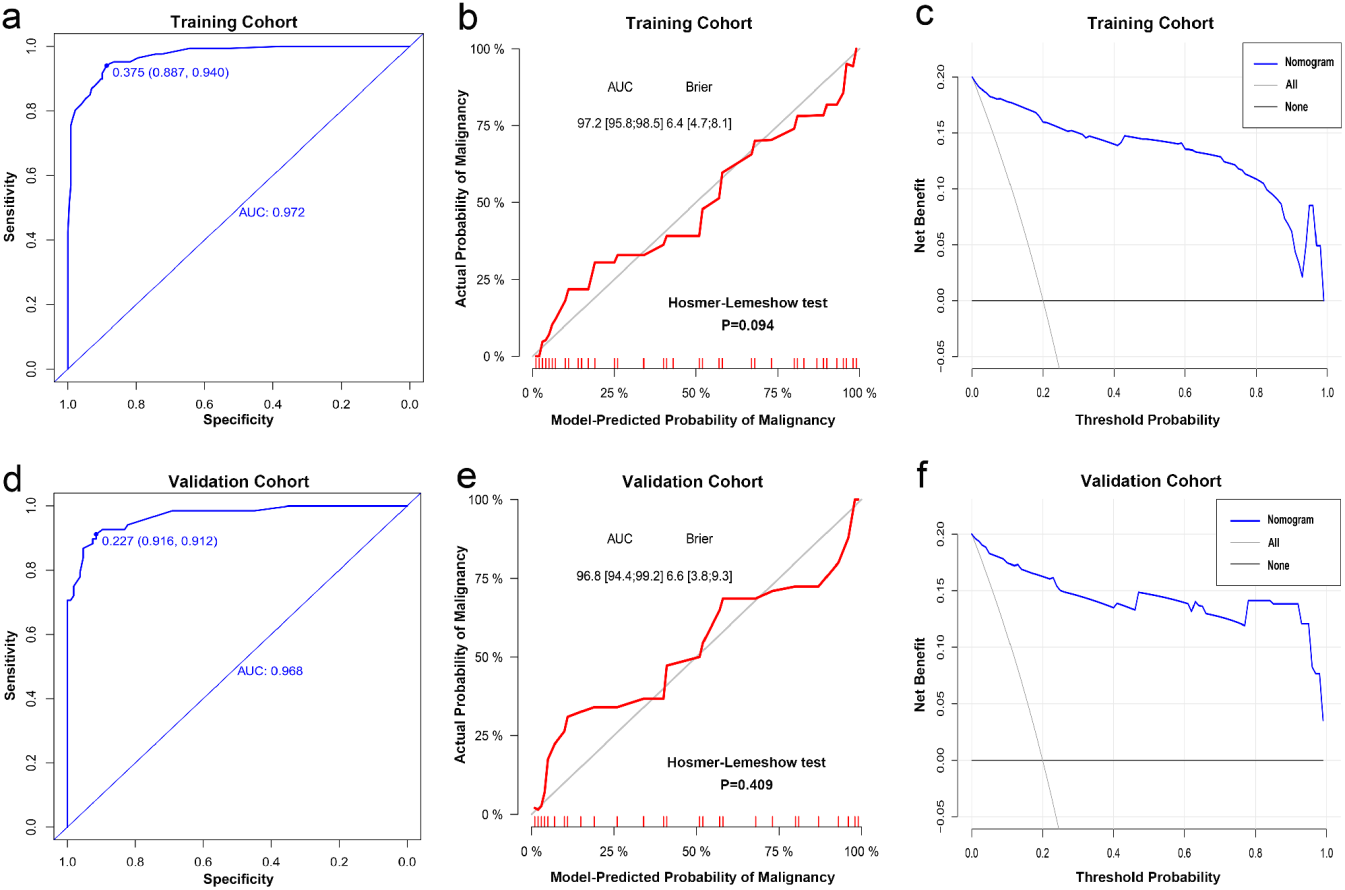
Predictive performance of the nomogram in the training and validation cohorts. (**a**) and (**d**) ROC curves of the nomogram in the training and validation cohorts; (**b**) and (**e**) Calibration curves of the nomogram in the training and validation cohorts. Nomogram-predicted probability of malignant thyroid nodules is on the X-axis; observed malignant thyroid nodules are shown on the Y-axis; and the grey solid line represents a perfect prediction. (**c**) and (**f**) Decision curves of the nomogram for predicting the probability of malignant thyroid nodules in the training and validation cohorts. The horizontal and vertical axes represent the threshold probability and net benefit, respectively. The blue line is the expected net beneft of per patient based on the predictive nomogram

The results of the multivariate logistic regression analysis were used to construct the nomogram for predicting malignant thyroid nodules, as depicted in Fig. 2. The AUCs of the nomogram in the training cohort and validation cohort were 0.972 (95% CI: 0.958–0.985) and 0.968 (95% CI: 0.944–0.992), respectively (Fig. 3a and d). The calibration curve (Fig. 3b and e) and Hosmer-Lemeshow test statistic (*P* = 0.094 and 0.409) demonstrated excellent calibration. Furthermore, the DCA analysis (Fig. 3c and f) indicated a larger overall net benefit for the nomogram.

### MRI-based risk scoring system

**Fig. 4:**
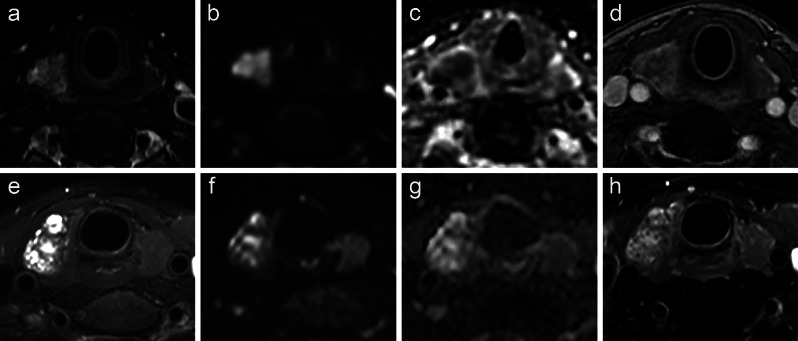
A 35-year-old female with a 1.8 cm papillary thyroid carcinoma in the right lobe of the thyroid gland (**a**, **b**, **c** and **d**) and A 47-year-old female with a 2 cm nodular goiter in the right lobe of the thyroid gland (**e**, **f**, **g** and **h**). Axial T2-weighted imaging (**a**) shows a striped low signal area; Axial diffusion-weighted imaging (DWI) (**b**) and apparent diffusion coefficient (ADC) map (**c**) show restricted diffusion of the lesion (high signal intensity on DWI, low signal intensity on ADC); The delayed phase contrast-enhanced T1-weighted imaging (**d**) reveals a greater enhancement in the peripheral area compared to the central area of the lesion, with a blurred border between them, which corresponds to the reversed halo sign in delay phase. The characteristics of the lesion (**a**, **b**, **c**, and **d**) including age < 48, low signal intensity on T2WI, restricted diffusion, and the presence of the reversed halo sign in delay phase, are consistent with an MRI risk scoring system score of 26 and indicate a determination of malignancy. Axial T2-weighted imaging (**e**) show a striped low signal area; Axial DWI (**f**) and ADC map (**g**) show high signal intensity of lesion; The delayed phase contrast-enhanced T1-weighted imaging (**h**) shows heterogeneous enhancement of lesion. The characteristics of the lesion (**e**, **f**, **g** and **h**) including age < 48 and low signal intensity on T2WI, are consistent with an MRI risk scoring system score of 6 and indicate a determination of benignity

The β coefficients corresponding for age, low signal intensity on T2WI, diffusion restriction, reversed halo sign in delay phase, cystic degeneration and wash-out pattern were 0.672, 1.367、3.677、2.958、1.490 and 1.112, respectively, and were subsequently assigned point values of 2, 4, 11, 9, 4, and 3 to developed the RSS. The optimal threshold for the RSS was 12 points. The AUCs for distinguishing between benign and malignant nodules were 0.914 (95%CI = 0.882–0.945) in the training cohort and 0.911 (95%CI = 0.880–0.962) in the validation cohort (Figure S1). The performance of the RSS is further elaborated in Table S5. Figure 4 depicts representative MRI images of both benign and malignant thyroid nodules.

### MRI-based prediction model

**Fig. 5:**
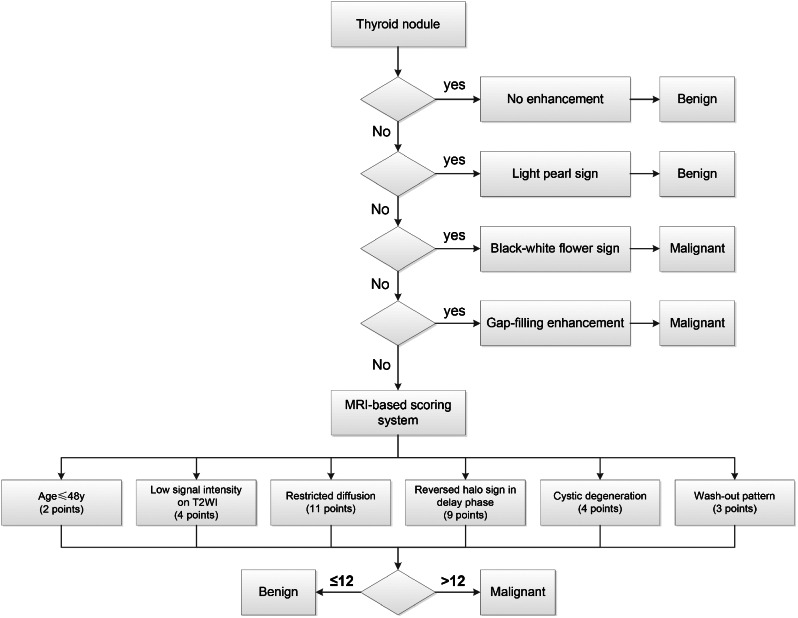
The MRI morphological features-based model for predicting benign and malignant thyroid nodules

**Fig. 6:**
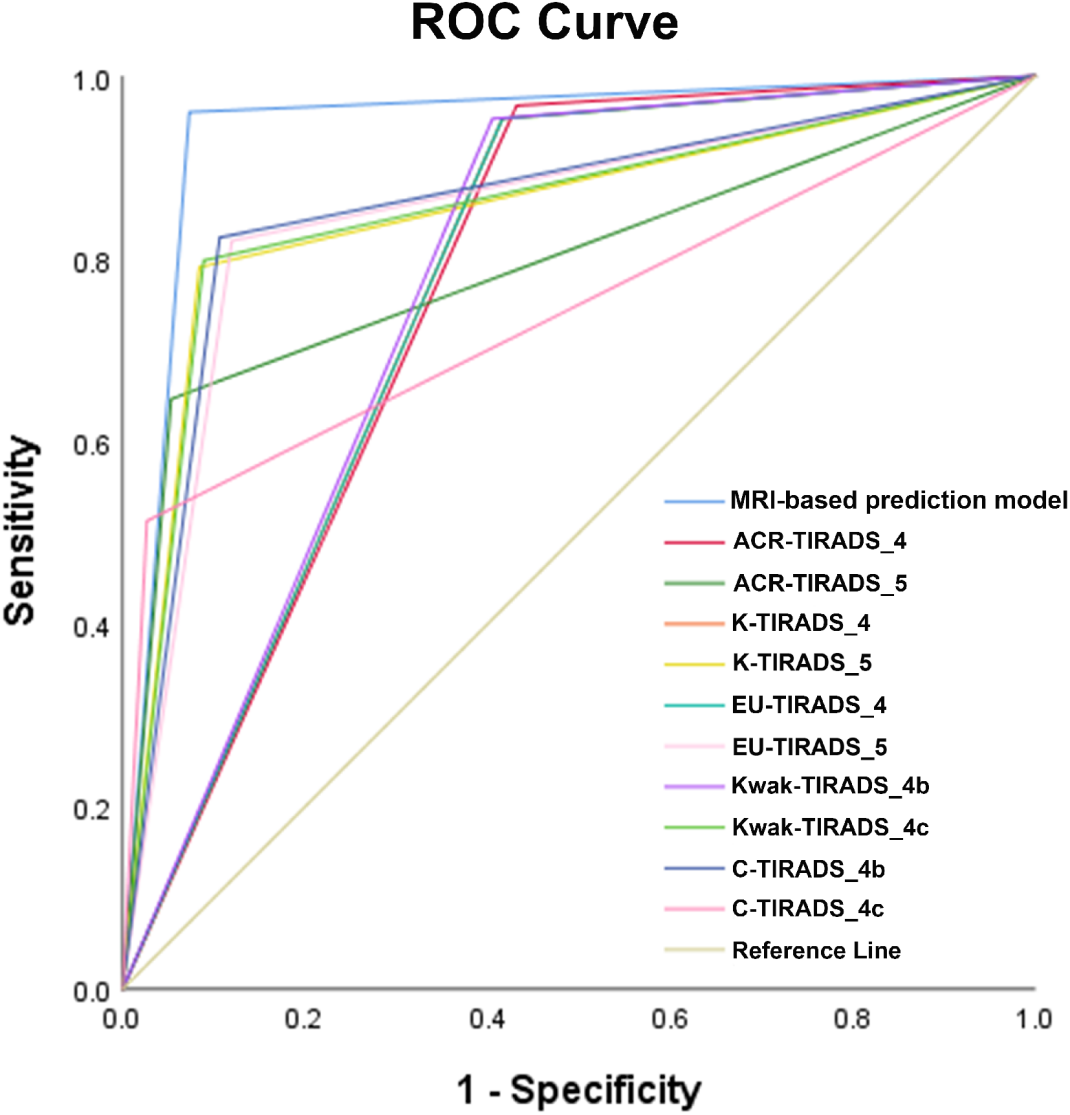
The receiver operating characteristic (ROC) curves of the MRI-based prediction model and TI-RADSs at various cut-off values for distinguishing between benign and malignant thyroid nodules. Abbreviation: ACR-TIRADS, American College of Radiology Thyroid Imaging Reporting and Data System; K-TIRADS, Korean-TIRADS; EU-TIRADS, European-TIRADS; C-TIRADS, Chinese-TIRADS

The construction of the MRI-based prediction model is depicted in Fig. 5. The diagnostic performance of the MRI-based prediction model and TI-RADSs is presented in Table 4. The MRI-based prediction model exhibited a larger AUC value of 0.947 (Delong test: *P* < 0.05), surpassing the AUC values of the other TI-RADSs (ranging from 0.747 to 0.858) as illustrated in Fig. 6. The sensitivity, specificity, accuracy, PPV, and NPV of the MRI-based prediction model were 96.0%, 93.4%, 94.4%, and 89.9%, respectively. The MRI-based prediction model demonstrated relatively superior specificity and NPV compared to the other five TI-RADSs, as well as improved predictive performance (NRI > 0, *P* < 0.05).

**Table 4:** Comparison of diagnostic performance of MRI-based prediction model and five ultrasonography-based risk stratification systems

Model (Cutoff score)	Sensitivity	Specificity	Accuracy	PPV	NPV	AUC	P*	NRI	P^#^
MRI-based prediction model	96.0	93.4	94.4	89.9	97.5	0.947			
ACR-TIRADS (≥ category 4)	96.8	56.9	72.1	57.9	96.6	0.768	<0.001	22.36	<0.001
ACR-TIRADS (≥ category 5)	64.6	94.7	83.3	88.2	81.4	0.797	<0.001	11.11	<0.001
K-TIRADS (≥ category 4)	95.3	58.4	72.4	58.4	95.3	0.769	<0.001	21.95	<0.001
K-TIRADS (≥ category 5)	79.1	91.6	86.8	85.2	87.7	0.853	<0.001	7.54	<0.001
EU-TIRADS (≥ category 4)	95.3	58.4	72.4	58.4	95.3	0.769	<0.001	21.95	<0.001
EU-TIRADS (≥ category 5)	81.9	88.1	85.7	80.8	88.8	0.850	<0.001	8.64	<0.001
Kwak-TIRADS (≥ category 4b)	95.3	59.5	73.1	59.1	95.4	0.774	<0.001	21.26	<0.001
Kwak-TIRADS (≥ category 4c)	79.8	91.2	86.8	84.7	88.0	0.855	<0.001	7.54	<0.001
C-TIRADS (≥ category 4b)	82.3	89.4	86.7	82.6	89.2	0.858	<0.001	7.68	<0.001
C-TIRADS (≥ category 4c)	51.3	97.3	79.9	92.2	76.5	0.743	<0.001	14.54	<0.001

Data are presented as percentages with the exception of the results for AUC. Abbreviation: PPV positive predictive value; NPV negative predictive value; AUC area under the curve; NRI Net reclassification index; TIRADS Thyroid Imaging Reporting and Data System; ACR American College of Radiology; *P values were assessed using Delong tests; ^#^the P value for NRI

*P values were assessed using Delong tests; ^#^the P value for NRI

Table 5 presents a comprehensive analysis of the diagnostic accuracy of the MRI-based prediction model across different subgroups. The performance of the MRI-based prediction model was notably strong in both unifocal and multifocal nodules, with particularly high sensitivity (96.1%) and specificity (95.7%) observed in multifocal nodules. Furthermore, the sensitivity and specificity of the prediction model in the presence or absence of Hashimoto’s thyroiditis were 94.6% and 96.8%, and 94.7% and 92.6%, respectively. The MRI-based prediction model demonstrated a sensitivity of 96.5%, 97.4%, and 60% for nodules measuring 0.5 to 1 cm, 1 to 4 cm, and larger than 4 cm, respectively, with corresponding specificities of 87.0%, 95.3%, and 95.1%. Notably, among nodules larger than 4 cm, a total of 8 malignant nodules were missed diagnosis, comprising seven follicular thyroid carcinomas and one papillary thyroid carcinoma.

**Table 5:** Subgroup analysis of MRI-based prediction model

Subgroup	N (%)	Sensitivity (%)	Specificity (%)	Accuracy (%)	PPV (%)	NPV (%)
Number						
Unifocal nodules subgroup	252(30.5)	93.2	81.1	88.9	89.9	86.9
Multifocal nodules subgroup	573(69.5)	96.1	95.7	95.8	89.2	98.5
Hashimoto′s thyroiditis						
Hashimoto′s thyroiditis subgroup	137(16.6)	94.6	96.8	95.6	97.2	93.8
Non-Hashimoto′s thyroiditis subgroup	688(83.4)	94.7	92.6	93.3	87.5	96.9
Size						
Small nodules subgroup (0.5-1 cm)	272 (33.0)	96.5	87.0	91.9	88.9	95.8
Medium nodules subgroup (1-4 cm)	452 (54.8)	97.4	95.3	96.0	91.6	98.6
Large nodules subgroup (≥4 cm)	101 (12.2)	60.0	95.1	88.1	75.0	90.6

Abbreviation: N, number; PPV, positive predictive value; NPV, negative predictive value; AUC, area under the curve

## Discussion

This study presented findings indicating that age, low signal intensity on T2WI, restricted diffusion, reversed halo sign in delay phase, cystic degeneration, and wash-out pattern were independent predictors for malignant thyroid nodules. The MRI-based nomogram was developed using these predictors showed satisfactory predictive ability and calibration in both training and validation cohorts. In addition, the MRI-based prediction model had superior specificity and NPV along with higher sensitivity compared to the other five TI-RADS, which improved the overall integrated prediction performance.

There have been a number of TI-RADSs that were developed as a result of previous studies using US to differentiate benign and malignant thyroid nodules. Kim DH et al. [11] found that ACR-TIRADS, K-TIRADS, and EU-TIRADS 4 and 5 category thyroid nodules had a good sensitivity greater than 90% in the meta-analysis. Their specificity, however, remained relatively low, with K-TIRADS having the highest specificity at 61%, followed by ACR-TIRADS (49%) and EU-TIRADS (48%). The suboptimal accuracy of thyroid cancer diagnosis has resulted in an increase in unnecessary FNA, which are both invasive and yield nondiagnostic outcomes in approximately 25% of cases [21]. This research study evaluated the performance of C-TIRADS, using a cut-off value of 4c category, and ACR-TIRADS, with a cut-off value of 5 category, in terms of specificity. C-TIRADS exhibited a specificity of 94.7%, while ACR-TIRADS demonstrated a specificity of 97.3%. However, both approaches sacrificed a significant amount of sensitivity, with values of only 51.3% and 64.6% respectively. In contrast, the MRI-based prediction model had the highest specificity of 93.4% while maintaining high sensitivity.

Risk stratification systems for thyroid nodules at ultrasound were frequently complicated and subject to low specificity and inadequate interobserver agreement. It is necessary to continuously improve these systems in order to minimize the unnecessary FNA. Wildman Tobriner et al. [22] showed that an artificial intelligence-optimized TI-RADS can moderately improve specificity and sensitivity compared to TI-RADS. In recent years, there has been a gradual increase in the research of MRI for diagnosing both benign and malignant thyroid nodules [23–27] and predicting the preoperative aggressiveness of papillary thyroid carcinoma [28–34], demonstrating promising application prospects. The ongoing advancements in MRI technology, as a functional medical imaging modality, warrant continuous exploration of its potential in diagnosing and evaluating thyroid nodules, ultimately leading to the development of its clinical application. Consequently, it becomes necessary to establish a prediction model for distinguishing between benign and malignant thyroid nodules based on MRI.

The predictive model developed in this study for distinguishing between benign and malignant thyroid nodules using MRI exhibited robust diagnostic efficacy. Integration of this model with the ultrasound TI-RADS grading system had the potential to improve diagnostic and treatment strategies for thyroid nodules. In clinical practice, patients categorized as TI-RADS category 4 or 5 may receive follow-up if the MRI-based predictive model suggests a benign nature, thereby avoiding unnecessary FNA. Conversely, if the model indicates malignancy, FNA and surgical intervention are recommended. However, further research was necessary to validate the effectiveness of the MRI-based prediction model in enhancing the ultrasound TI-RADS grading system. Subgroup analysis revealed a decreased sensitivity of the MRI-based prediction model for thyroid nodules larger than 4 cm, with missed diagnoses of 7 cases of thyroid follicular carcinoma and 1 case of thyroid papillary carcinoma. These findings indicated a limited diagnostic efficacy of the model for follicular thyroid neoplasms (FTNs) larger than 4 cm. Lin et al. [35] highlighted the ineffectiveness of various TIRADSs in managing FTNs, as evidenced by the high percentage (65.3 to 93.1%) of patients subjected to unnecessary FNA. This highlighted the importance of establishing a tailored stratification system for FTNs. Consequently, it was imperative to create MRI-based predictive models specifically designed for FTNs.

In this study, all nodules that exhibit non-enhancement and the light pearl sign were confirmed to be benign. It was observed that these two features were identified as specific characteristics indicated benignity. The presence of the black-white flower sign and gap-filling enhancement were found to be specific features associated with malignant nodules. The black-white flower sign was defined as petaloid and cerebrospinal fluid-like high signal on T2WI with an irregular apparent low signal in the centre of the lesion. From a pathological perspective, this sign corresponds to the stromal appearance, where it segregated follicular cells into irregular petal-like structures that aggregate in the central area of the lesion. The stroma contains few fluid content, resulting in low signal intensity on T2WI, whereas the presence of numerous follicular cells with higher fluid content contributes to the high signal on T2WI. Gap-filling enhancement was defined as a lesion located in the perithyroid region with a progressive enhancement pattern, with disruption of the contour line in the early phases and an intact contour line in the delayed phases. In the early phases of enhancement, interrupted thyroid contour was a sign of involvement of the thyroid envelope by the stromal component of the lesion [36], whereas in the delayed phase this stromal enhancement leads to filling of the interrupted envelope.

In the RSS, restricted diffusion and reversed halo sign in delay phase were the two independent predictors with the highest scores of 11 and 9 points. DWI has been widely employed in the field of oncology for the purposes of diagnosing, monitoring, and prognosticating malignancies [37]. The apparent diffusion coefficient (ADC) has been established as a valuable tool in the differentiation of benign and malignant thyroid nodules [38–40]. Restricted diffusion was defined by the presence of a solid component within the lesion, which manifested as high signal intensity on DWI and low signal intensity on ADC. The assessment of restricted diffusion provided a direct and pragmatic approach in contrast to the utilization of quantitative ADC values. The reversed halo sign in delay phase was defined by the wash-out pattern of the central portion of the lesion, continuous enhancement in the peripheral area relative to the central part during the delay phase, and a blurred border. This finding aligns with a previous study conducted by Wang et al. [41]. The interpretation implied that the central region of the tumor exhibits active proliferation of neoplastic cells, whereas the peripheral area primarily consisted of connective tissue with a profusion of tomur stroma, leading to sustained enhancement in the delay phase.

This study exhibited several limitations. Firstly, it adopted a retrospective design, thereby introducing an inherent selection bias, as it exclusively includes cases that underwent surgery intervention for pathological examination. Consequently, the exclusion of nodules selected for follow-up after FNA or those deemed too small for FNA may have influenced the outcomes. Secondly, the study did not encompass nodules smaller than 5 mm due to the limitation imposed by MRI imaging techniques. Thirdly, the qualitative parameters selected in this study possessed a certain degree of subjectivity. Although quantitative parameters were considered objective, their accuracy can be affected by various factors such as equipment, parameters, and measurement methods in clinical practice. In contrast, qualitative indicators provided convenience in clinical settings. Lastly, this study was limited to a single-center, and incorporating multi-center cases would enhance the validation of the MRI-based prediction model.

In conclusion, the utilization of MRI morphological features in the prediction model for benign and malignant thyroid nodules demonstrated a notable diagnostic efficacy, thereby establishing a reliable basis for clinical diagnosis and treatment decision-making. Moreover, the incorporation of MRI morphological features holds promise in enhancing the diagnostic accuracy of TI-RADS.

### Electronic supplementary material

Below is the link to the electronic supplementary material.

Supplementary Material 1

## Data Availability

The data sets generated and/or analyzed in the current study were not made public because patients’ personal information was included. Available from the corresponding author upon reasonable request.

## Abbreviations

US: Ultrasonography
ACR: American College of Radiology
TI-RADS: Thyroid Imaging Reporting and Data System
K-TIRADS: Korean Thyroid Imaging Reporting and Data System
EU-TIRADS: European Thyroid Imaging Reporting and Data System
C-TIRADS: Chinese Thyroid Imaging Reporting and Data System
FNA: Fine-needle aspiration
T1WI: T1 weighted imaging
T2WI: T2 weighted imaging
DWI: Diffusion-weighted imaging
MCE-T1WI: multiphasic contrast-enhanced T1WI
ROC: Receiver operating characteristic
DCA: Decision curve analysis
RSS: Risk scoring system
PPV: Positive predictive value
NPV: Negative predictive value
NRI: Net reclassification index
SD: Standard deviation
CI: Confidence intervals
ADC: Apparent diffusion coefficient
