# Supplementary material for: Prediction model based on MRI morphological features for distinguishing benign and malignant thyroid nodules
Source: BMC Cancer. 2024 Feb 23;24:256. doi: 10.1186/s12885-024-11995-3 (PMC10885392; doi:10.1186/s12885-024-11995-3)
Supplement: Supplementary file 1 — Supplementary Material 1 [file 12885_2024_11995_MOESM1_ESM.docx]

**Supplementary Material**

**MRI parameter definition and diagram**

1. **Black-white flower sign** was defined as the lesion with high signal similar to cerebrospinal fluid signal with irregular petal shaped, and irregular and obvious low signal in the center of the lesion on T2WI, **which was indicated in the figures as follows**.

**
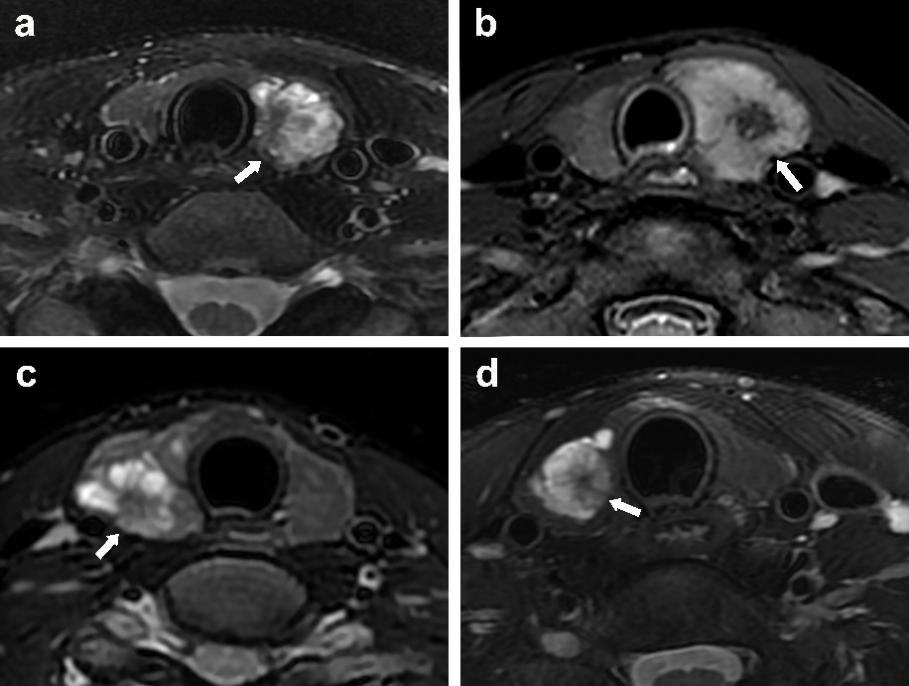
**

The white arrow refers to the black-white flower sign on T2WI image from four different patients.

1. **Gap-filling enhancement** was defined as the lesion located at the peripheral area of thyroid with thyroid contour line interruption in the early phase of multiphasic contrast-enhanced MRI had a progressive enhancement pattern, and then the thyroid contour line was complete in the delay phase, **which was indicated in the figures as follows**.

**
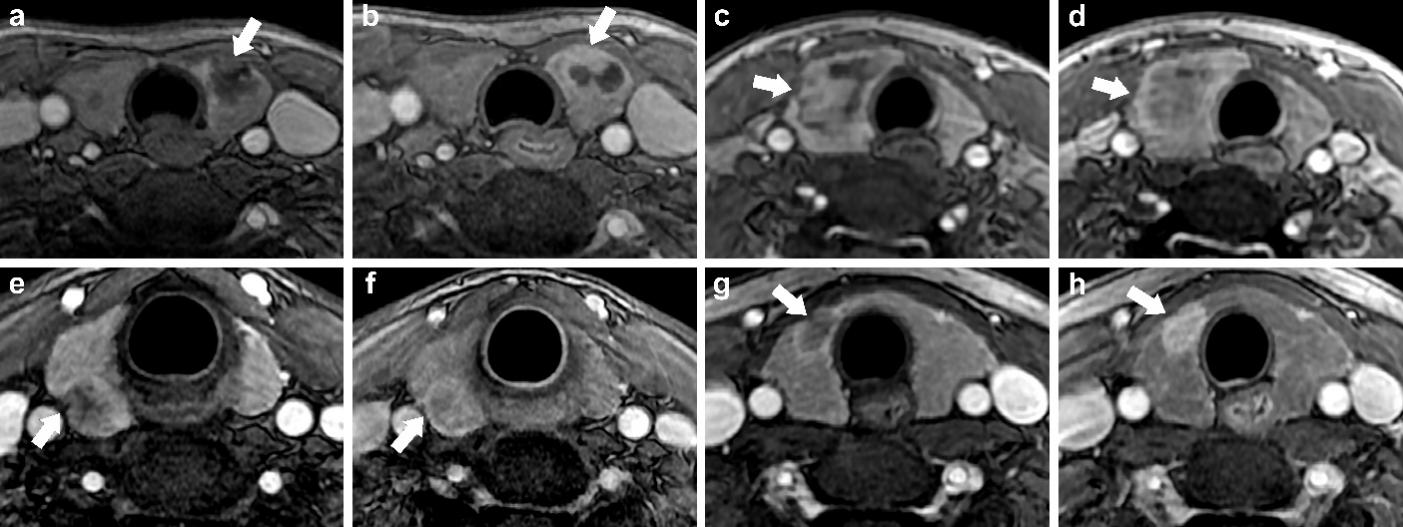
**

The early phase (a, c, e, g) and delay phase (b, d, f, h) of multiphasic contrast-enhanced MRI show Gap-filling enhancement (white arrows), and the images (a, b), (c, d), (e, f) and (g, h) were obtained from the same patients.

1. **Light pearl sign** was defined as the single or multiple nodular irregular lesions with hyperintense signal like cerebrospinal fluid on the wall of the black cystic lesions on T2WI, **which was indicated in the figures as follows**.

**
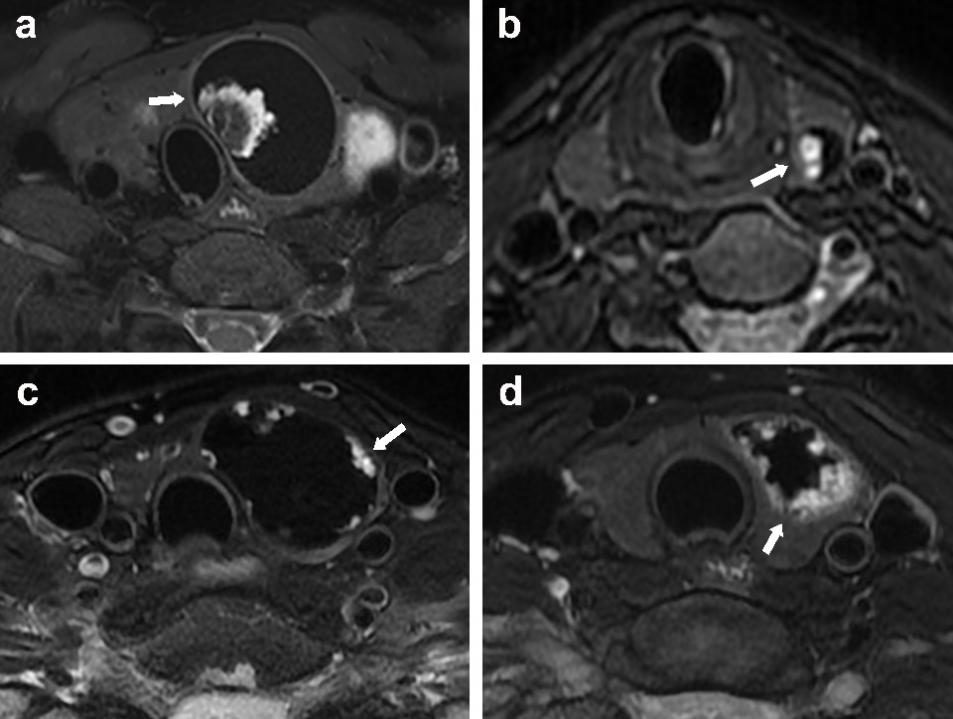
**

The white arrow refers to the light pearl sign on T2WI image from four different patients.

1. **No enhancement** was defined as no contrast enhancement for lesion in the early phase and delay phase of multiphasic contrast-enhanced MRI, **which was indicated in the figures as follows**.

**
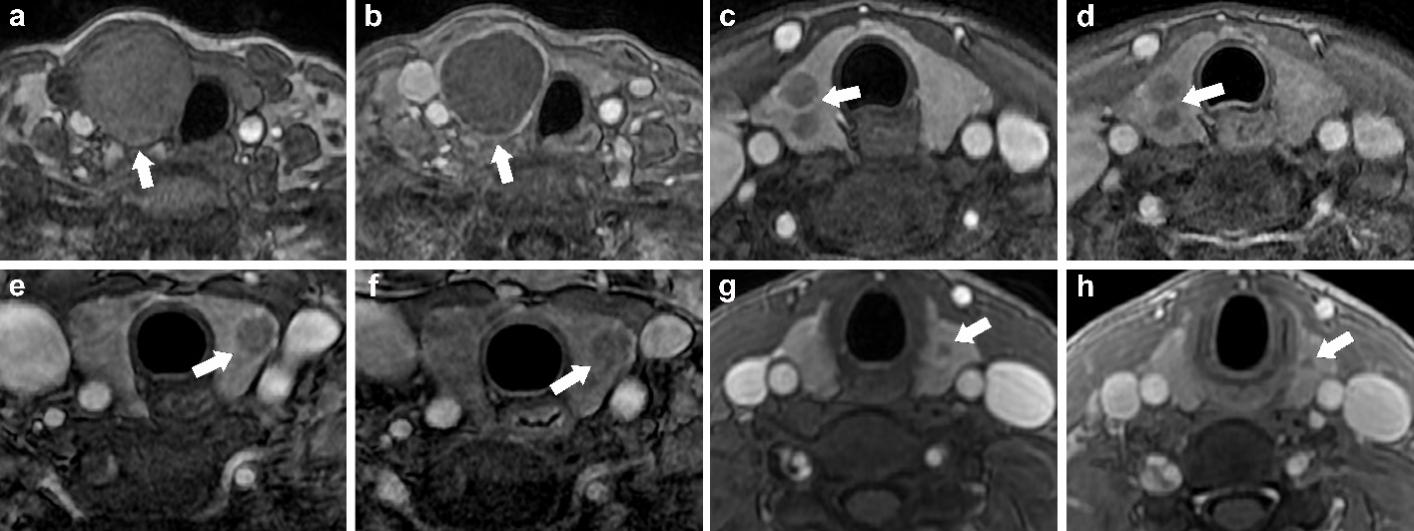
**

The images (a, b), (c, d), (e, f) and (g, h) were obtained from the same patients and showed no enhancement that are hypointense on pre-contrast T1WI (a) and no contrast enhancement in the early phase (c, e, g) and delay phase (b, d, f, h) (white arrows)

1. **High signal intensity on T2WI**

**The criteria for diagnosis:** high signal intensity on T2WI images can be seen in focal or diffuse area of thyroid nodule, similar to cerebrospinal fluid signal intensity. **Variable was indicated in the figures as follows:**

**
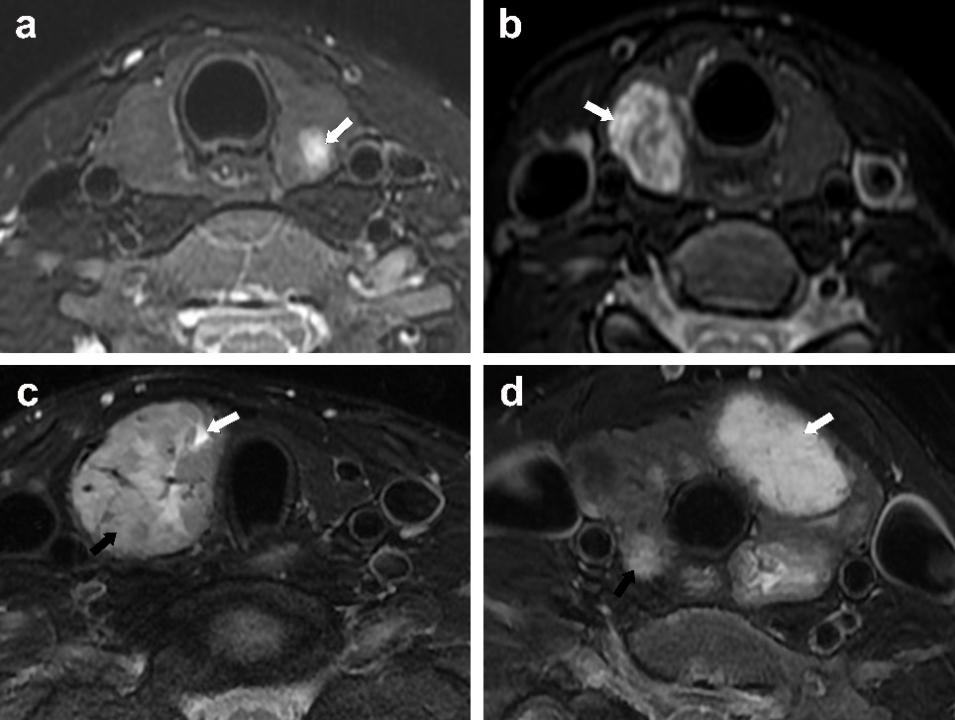
**

1. **High signal intensity on T1WI**

**The criteria for diagnosis:** high signal intensity on T1WI images can be seen in focal or diffuse area of thyroid nodule.**Variable was indicated in the figures as follows:**

**
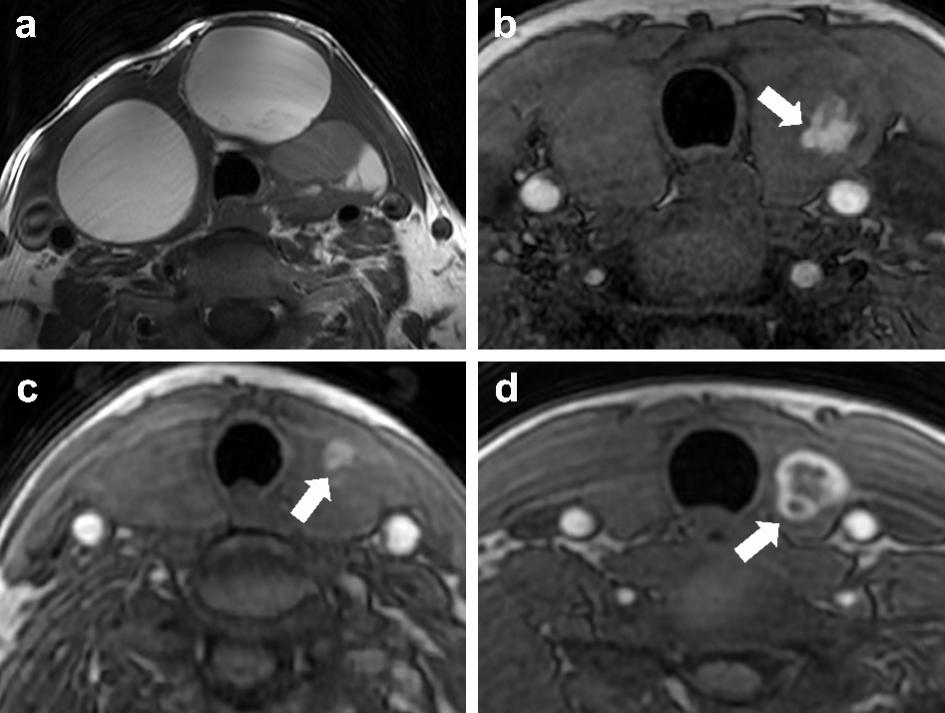
**

1. **Low signal intensity on T2WI**

**The criteria for diagnosis:** low signal intensity on T2WI images can be seen in focal or diffuse area of thyroid nodule. **Variable was indicated in the figures as follows:**

**
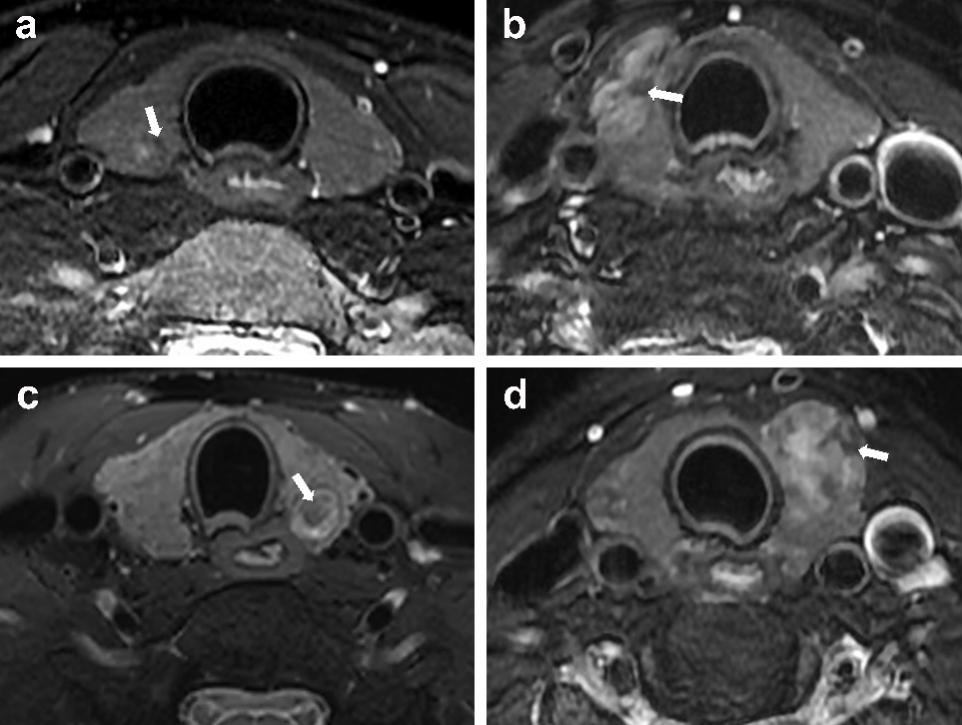
**

1. **Restricted diffusion** was identified as areas that were hyperintense or isointense on DWI images, hypointense on ADC maps and presented with enhancement, **which was indicated in the figures as follows**.

**
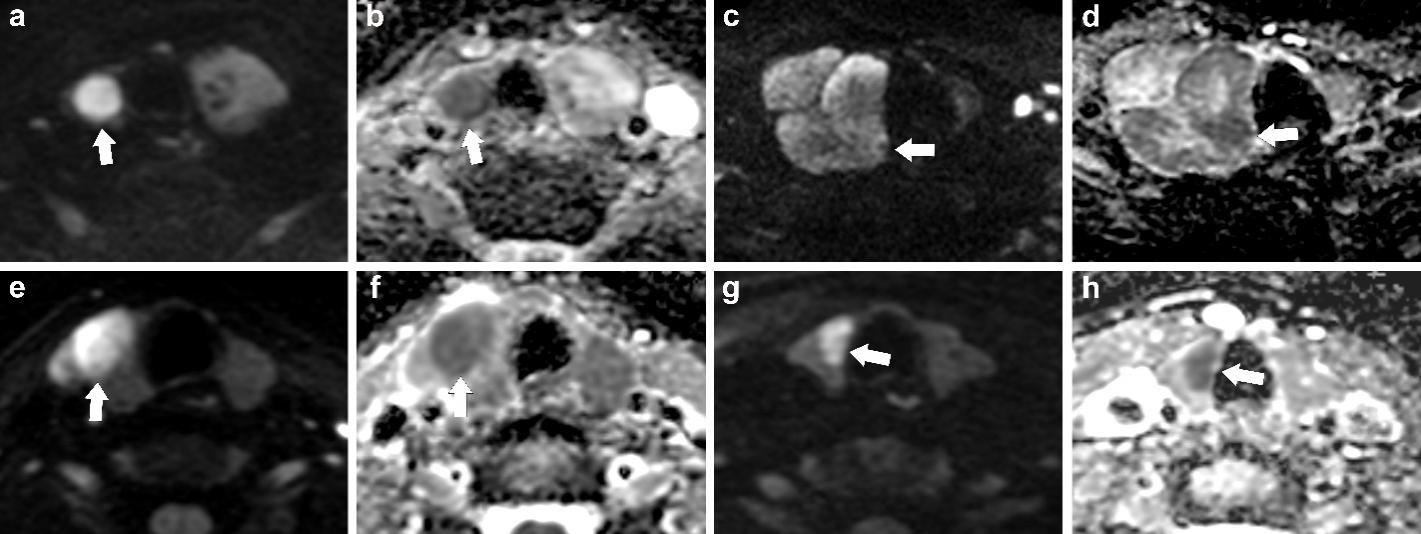
**

The images (a, b), (c, d), (e, f) and (g, h) were obtained from the same patients and showed restricted diffusion that are hyperintense on DWI (a, c, e, g) and hypointense on ADC (b, d, f, h) (white arrows)

1. **Reversed halo sign in delay phase** was defined as the enhancement pattern that the enhancement of the peripheral area of the lesion was greater than that of the central area in delay phase of multiphasic contrast-enhanced MRI, and the demarcation between area was blurred, **which was indicated in the figures as follows**.

**
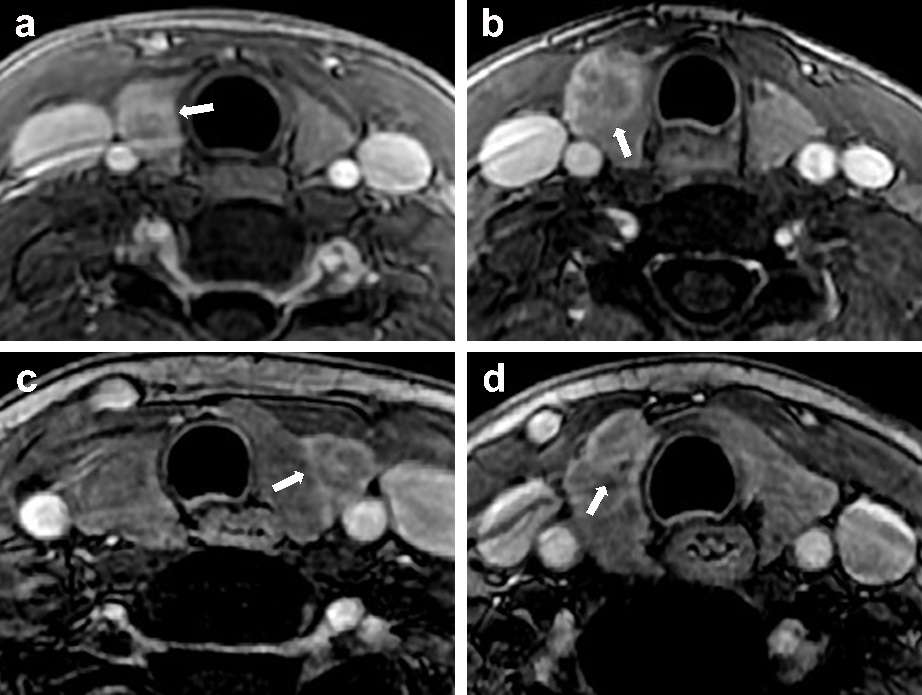
**

The white arrow refers to the reversed halo sign in delay phase from four different patients.

1. **Pseudocapsule** was defined as a peritumoral rim that showed enhancement in multiphasic contrast-enhanced MRI imaging, **which was indicated in the figures as follows**.


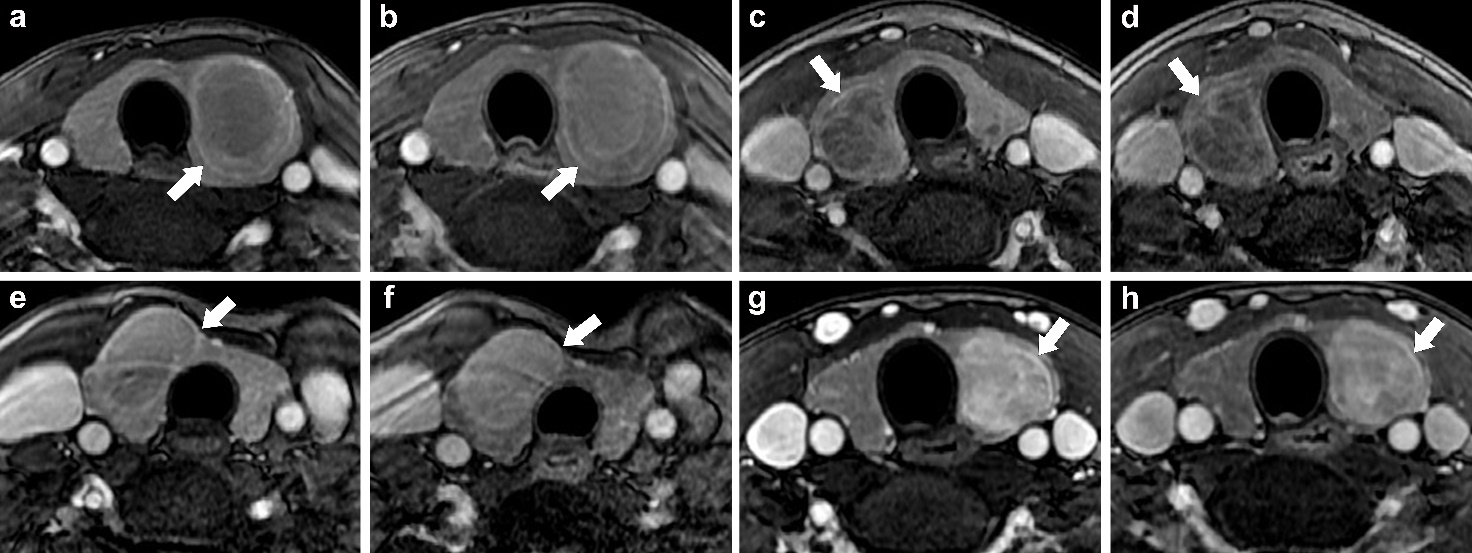


The early phase (a, c, e, g) and delay phase (b, d, f, h) of multiphasic contrast-enhanced MRI show pseudocapsule sign (white arrows), and the images (a, b), (c, d), (e, f) and (g, h) were obtained from the same patients.

1. **Fissure-filling enhancement** was defined as the irregular fissure that did not enhance in the early phase in the internal area of the lesion presented a progressive and filling enhancement pattern in the delay phase, **which was indicated in the figures as follows.**

**
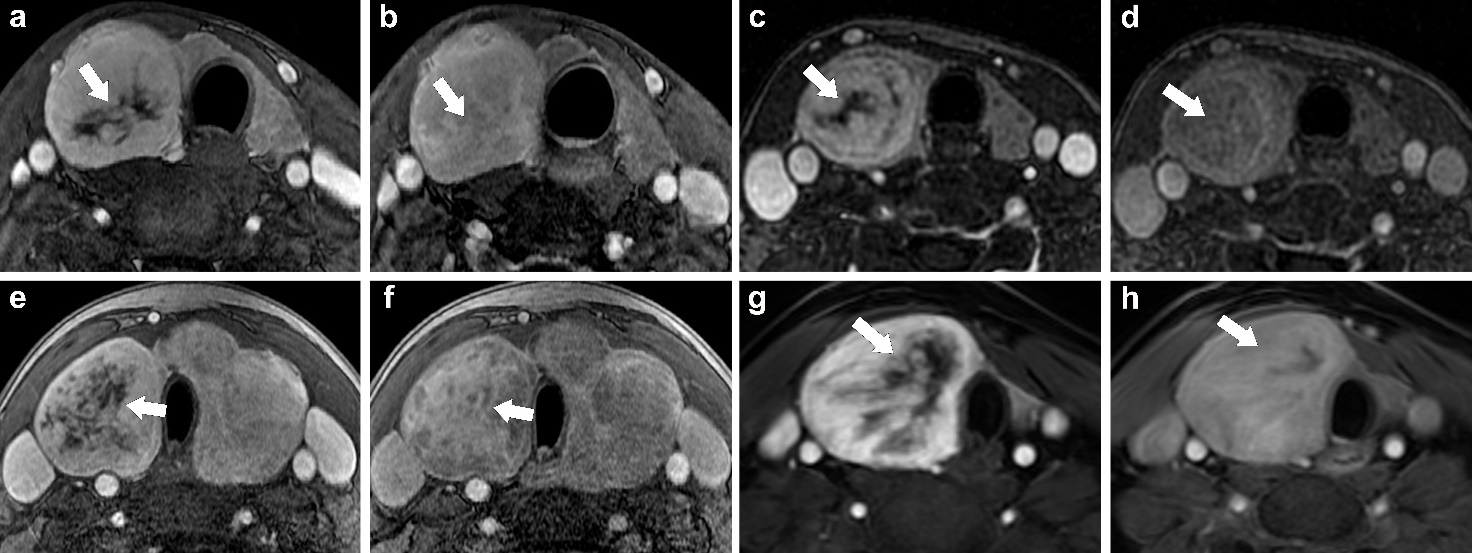
**

The early phase (a, c, e, g) and delay phase (b, d, f, h) of multiphasic contrast-enhanced MRI show fissure-filling enhancement (white arrows), and the images (a, b), (c, d), (e, f) and (g, h) were obtained from the same patients.

1. **Cystic degeneration** was identified as the area that was hypointense on pre-contrast T1-weighted images, markedly hyperintense on T2-weighted images and presented with non-enhancement, **which was indicated in the figures as follows**.


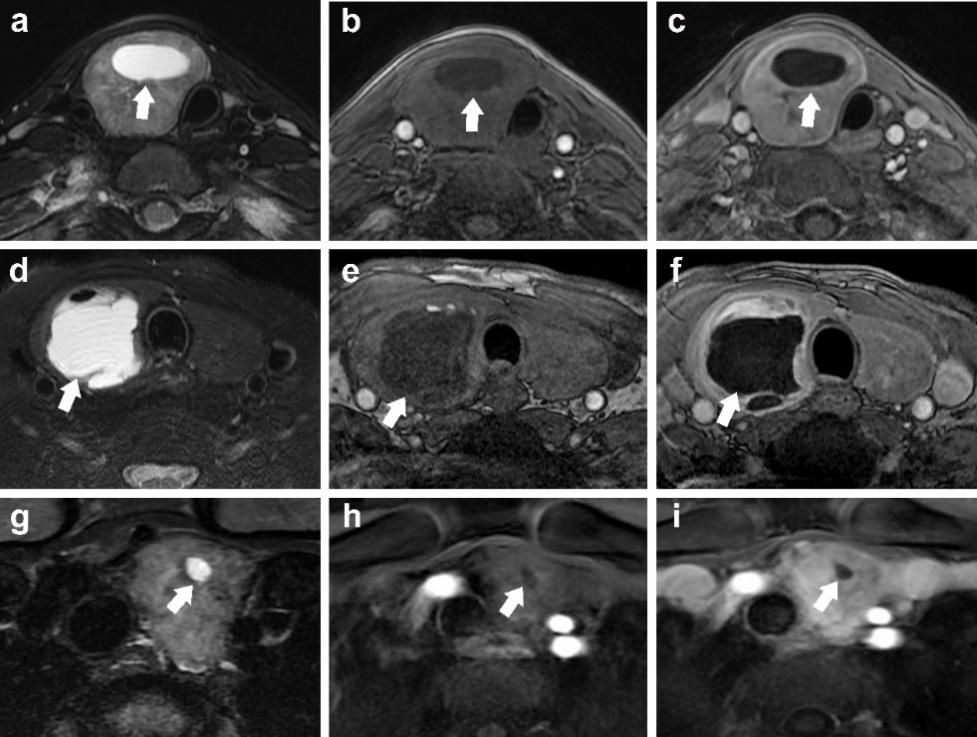


The images (a, b, c), (d, e, f) and (g, h, i) were obtained from the same patients and showed cystic degeneration (white arrows) that are markedly hyperintense on T2WI (a, d, g), hypointense on T1WI (b, e, h) and no enhancement was observed on the contrast-enhanced T1WI (c, f, i).

1. **hyperintense on T2WI with enhancement** was defined as high signal similar to cerebrospinal fluid signal on T2WI with enhancement in contrast-enhanced phase, **which was indicated in the figures as follows**.


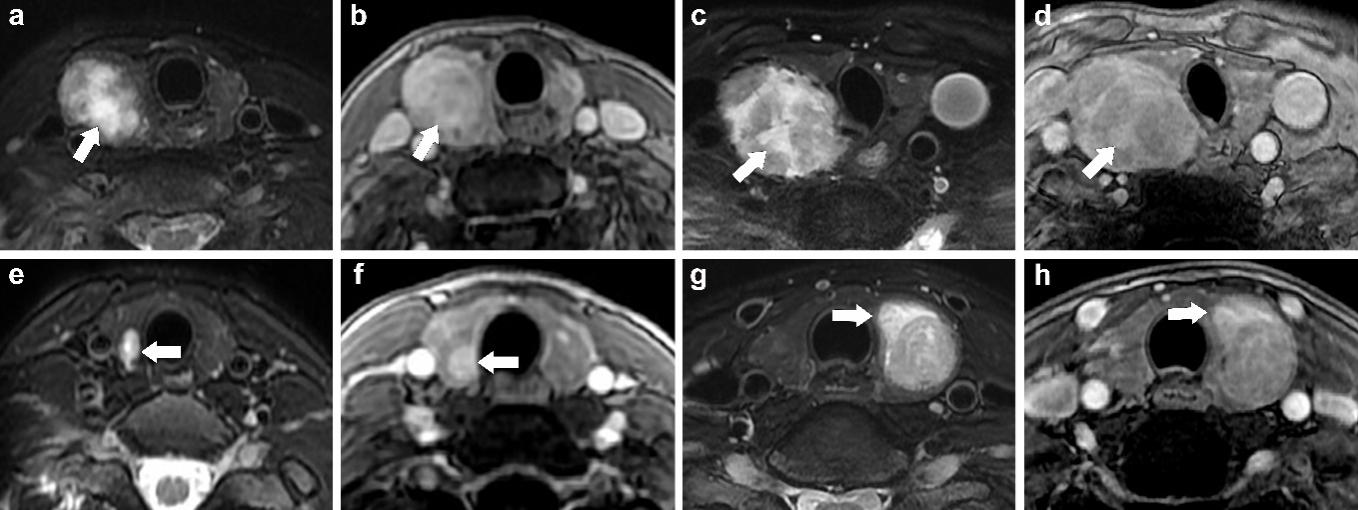


The images (a, b), (c, d), (e, f) and (g, h) were obtained from the same patients and showed hyperintense on T2WI with enhancement (white arrows) that are markedly hyperintense on T2WI (a, c, e, g) and enhancement was observed on the contrast-enhanced T1WI (b, d, f, h).

1. **Flow-void signal** was identified as multiple lines with no signal in the lesion on T2WI image, **which was indicated in the figures as follows**.

**
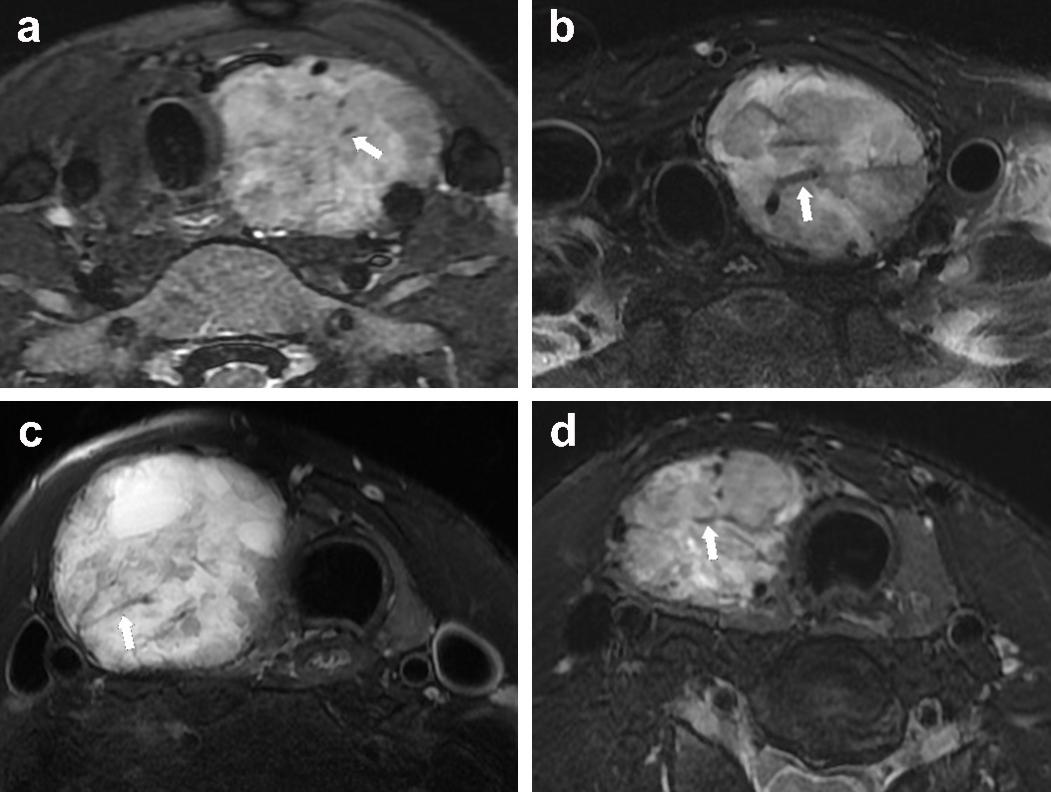
**

The white arrow refers to the flow-void signal from four different patients.

1. **Wash-out pattern** was identified as the enhancement of lesion in early phase of multiphasic contrast-enhanced, and the degree of enhancement in delay phase decreased, **which was indicated in the figures as follows**.

**
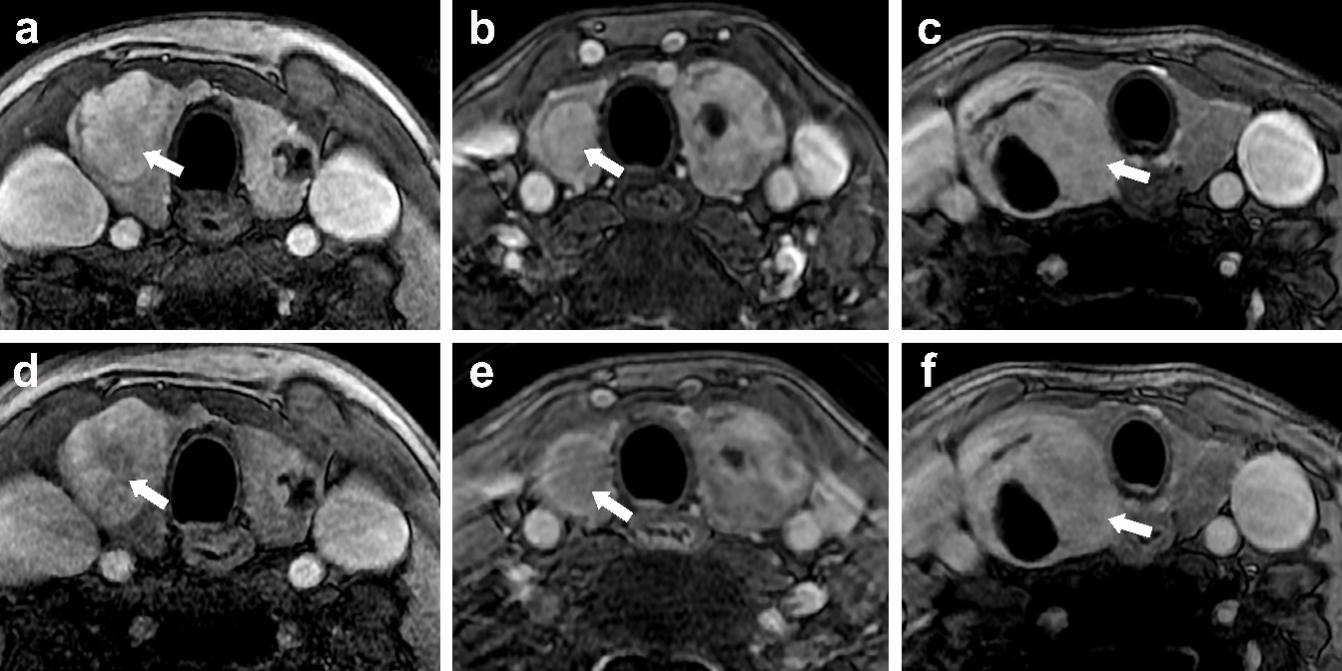
**

The early phase (a, b, c) and delay phase (d, e, f) of multiphasic contrast-enhanced MRI showed wash-out pattern (white arrows), and the images (a, d), (b, e) and (c, f) were obtained from the same patients.

1. **Hyperenhancement in early phase** was defined as the enhancement degree of lesion similar to that of the common carotid artery in the early phase of multiphasic contrast-enhanced MRI, **which was indicated in the figures as follows**.

**
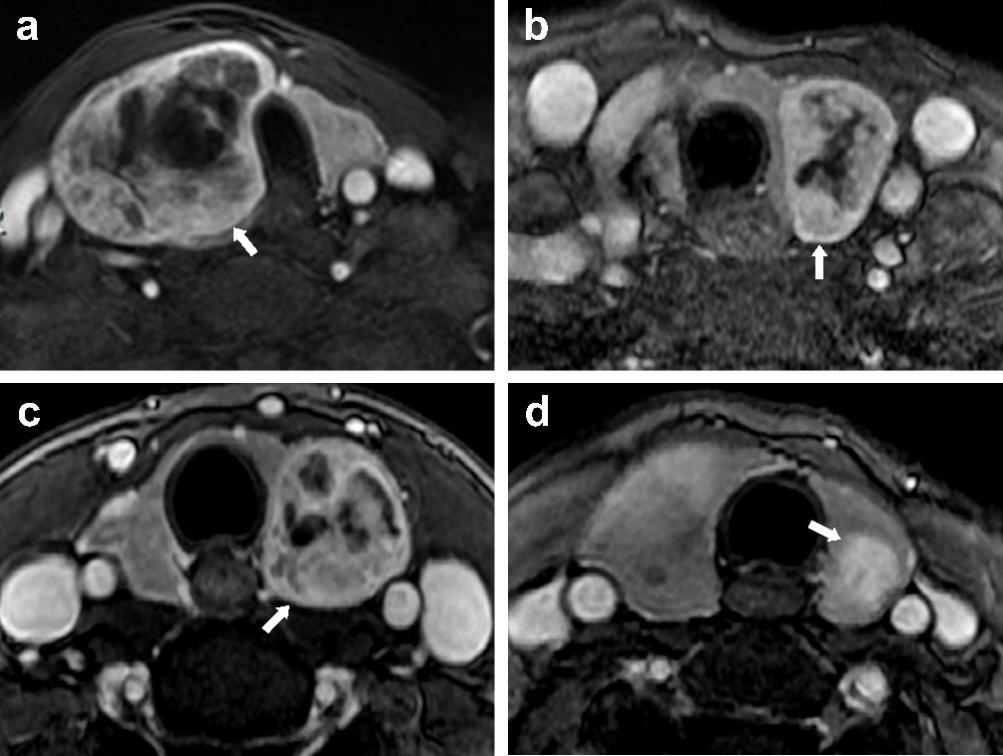
**

The white arrow refers to hyperenhancement in early phase from four different patients.

1. **Change of lesion size in multiphasic enhancement** was defined as changes in the size and morphology of lesion in the early phase and delay phase of multiphasic contrast-enhanced MRI, **which was indicated in the figures as follows**.


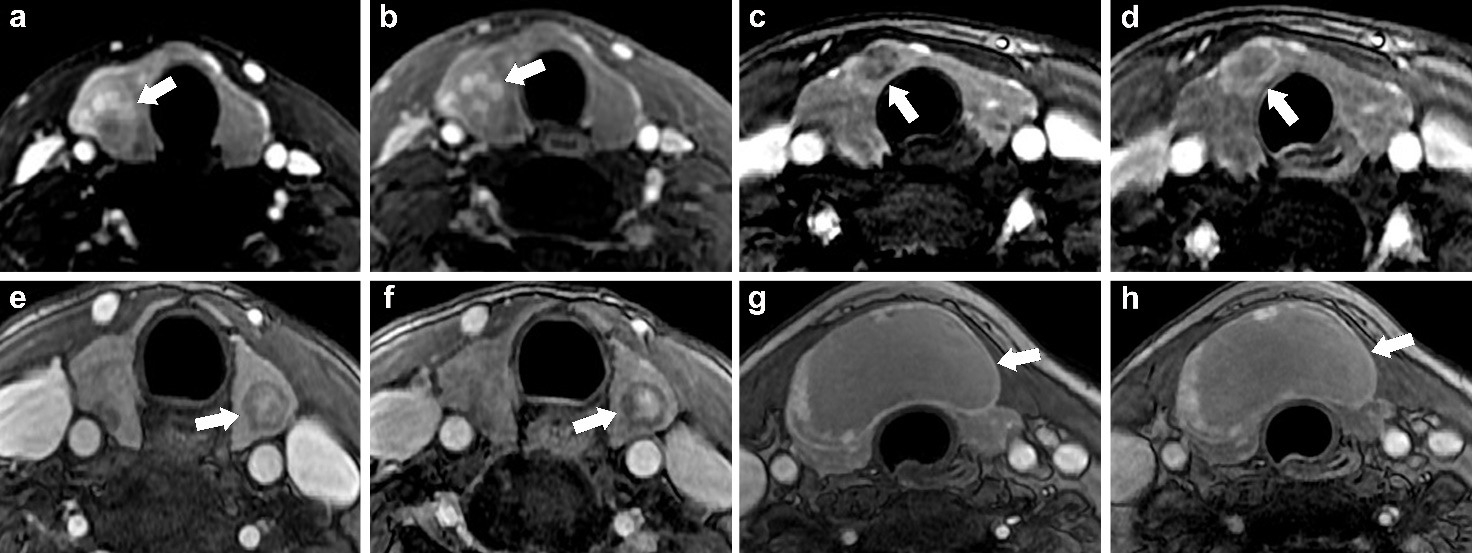


The images (a, b), (c, d), (e, f) and (g, h) were obtained from the same patients. The early phase (a, c, e, g) and delay phase (b, d, f, h) of multiphasic contrast-enhanced MRI showed change (a, b, c, d) and no change (e, f, g, h) of lesion in multiphasic enhancement.

**Table S1.** Parameters of MRI Sequence

|  | Plane | TR | TE | Slice thickness | Gap between slices | NEX | FOV | Matrix size | Sequence |
| --- | --- | --- | --- | --- | --- | --- | --- | --- | --- |
| T2WI with fat suppression | coronal | 1300 | 81.2 | 4 | 0.5 | 4 | 20 | 288×192 | FRFSE |
| T1WI | axial | 540 | 10.6 | 4 | 0.5 | 2 | 20 | 288×224 | FSE-XL |
| T2WI with fat suppression | axial | 3000 | 86.9 | 4 | 0.5 | 2 | 20 | 320×224 | FRFSE |
| DWI | axial | 6550 | 85 | 4 | 0.5 | 8 | 20 | 128×128 | SE-EPI |
| Contrast-enhanced T1WI | axial | 5.5 | 1.6 | 4 | -2 | 1 | 25 | 256×192 | FSPGR |
| Units |  | ms | ms | mm | mm |  | cm |  |  |

Abbreviations: TR, repetition time; TE, echo time; NEX, number of excitations; FOV, field of view; T2WI, T2-weighted image; T1WI, T1-weighted image; DWI, diffusion-weighted imaging; FRFSE, fast recovery fast spin echo; FSE, fast spin echo; SS-EPI, single shot echo-planar imaging; FSPGR, fast spoiled gradient echo.

**Table S2.** Pathological types

| Pathological types | N |
| --- | --- |
| Papillary thyroid carcinoma | 291 (35.3) |
| Follicular thyroid carcinoma | 17 (2.1) |
| Medullary thyroid carcinoma | 5 (0.6) |
| Undifferentiated carcinoma | 2 (0.2) |
| Nodular goiter | 355 (43.0) |
| Adenomatous goiter | 69 (8.4) |
| Adenoma | 47 (5.7) |
| Nodular hashimoto thyroiditis | 23 (2.8) |
| Subacute thyroiditis | 12 (1.5) |
| Other | 4 (0.5) |

Data are expressed as the number of nodules, with percentages in parentheses.

**Table S3.** Distribution of specific morphological features in benign and malignant nodules

| Characteristics | Benign (n=508) | Malignant（n=317) | P value | Kappa value |
| --- | --- | --- | --- | --- |
| No enhancement |  |  | <0.001* | 0.970 |
| Present | 123 (24.2) | 0 (0.0) |  |  |
| Absent | 385 (75.8） | 317（100.0) |  |  |
| Light pearl sign |  |  | <0.001* | 0.987 |
| Present | 47 (9.3) | 0 (0.0) |  |  |
| Absent | 461 (90.7) | 317 (100.0) |  |  |
| Black-white flower sign |  |  | <0.001* | 0.899 |
| Present | 0 (0.0) | 48 (15.1) |  |  |
| Absent | 508 (100) | 269 (84.9) |  |  |
| Gap-filling enhancement |  |  | <0.001* | 0.886 |
| Present | 0 (0.0) | 38 (12.0) |  |  |
| Absent | 508 (100.0) | 279 (88.0) |  |  |

Data are expressed as the number of nodules, with percentages in parentheses. *, P<0.05.

**Table S4.** TI-RADS classification results for 729 thyroid nodules

| TI-RADS | Class | Benign (n=452) | Malignant (n=277) | Total | Rate of malignant (%) | P value |
| --- | --- | --- | --- | --- | --- | --- |
| ACR-TIRADS |  |  |  |  |  | <0.001* |
|  | TI-RADS 2 | 5 | 0 | 5 | 0.00 |  |
|  | TI-RADS 3 | 253 | 8 | 261 | 3.07 |  |
|  | TI-RADS 4 | 171 | 89 | 260 | 34.23 |  |
|  | TI-RADS 5 | 23 | 180 | 203 | 88.67 |  |
| K-TIRADS |  |  |  |  |  | <0.001* |
|  | TI-RADS 2 | 4 | 0 | 4 | 0.00 |  |
|  | TI-RADS 3 | 263 | 13 | 273 | 4.76 |  |
|  | TI-RADS 4 | 150 | 45 | 195 | 23.08 |  |
|  | TI-RADS 5 | 38 | 219 | 257 | 85.21 |  |
| EU-TIRADS |  |  |  |  |  | <0.001* |
|  | TI-RADS 2 | 4 | 0 | 4 | 0.00 |  |
|  | TI-RADS 3 | 260 | 13 | 283 | 4.59 |  |
|  | TI-RADS 4 | 134 | 37 | 171 | 21.64 |  |
|  | TI-RADS 5 | 54 | 227 | 281 | 80.78 |  |
| Kwak-TIRADS |  |  |  |  |  | <0.001* |
|  | TI-RADS 2 | 4 | 0 | 4 | 0.00 |  |
|  | TI-RADS 3 | 217 | 9 | 226 | 3.98 |  |
|  | TI-RADS 4a | 48 | 4 | 52 | 7.69 |  |
|  | TI-RADS 4b | 143 | 43 | 186 | 23.12 |  |
|  | TI-RADS 4c | 39 | 204 | 243 | 83.95 |  |
|  | TI-RADS 5 | 1 | 17 | 18 | 94.44 |  |
| C-TIRADS |  |  |  |  |  | <0.001* |
|  | TI-RADS 2 | 13 | 0 | 13 | 0.00 |  |
|  | TI-RADS 3 | 208 | 11 | 219 | 5.02 |  |
|  | TI-RADS 4a | 183 | 38 | 221 | 17.19 |  |
|  | TI-RADS 4b | 36 | 86 | 122 | 70.49 |  |
|  | TI-RADS 4c | 11 | 125 | 136 | 91.91 |  |
|  | TI-RADS 5 | 1 | 17 | 18 | 94.44 |  |

[Abbreviation](javascript:;): ACR, American College of Radiology；TIRADS, Thyroid Imaging Reporting and Data System. **P*＜0.05.

**Table S5.** Performance of the MRI-based risk scoring system in the training and validation cohorts

| MRI-based RSS (Cutoff=12) | Sensitivity | Specificity | Accuracy | PPV | NPV | AUC |
| --- | --- | --- | --- | --- | --- | --- |
| Training cohort | 89.7 | 92.5 | 91.5 | 88.4 | 93.4 | 0.914 |
| Validation cohort | 94.0 | 88.7 | 90.9 | 85.8 | 95.3 | 0.911 |

[Abbreviation](javascript:;): PPV positive predictive value; NPV negative predictive value; AUC area under the curve; RSS risk score system.

**
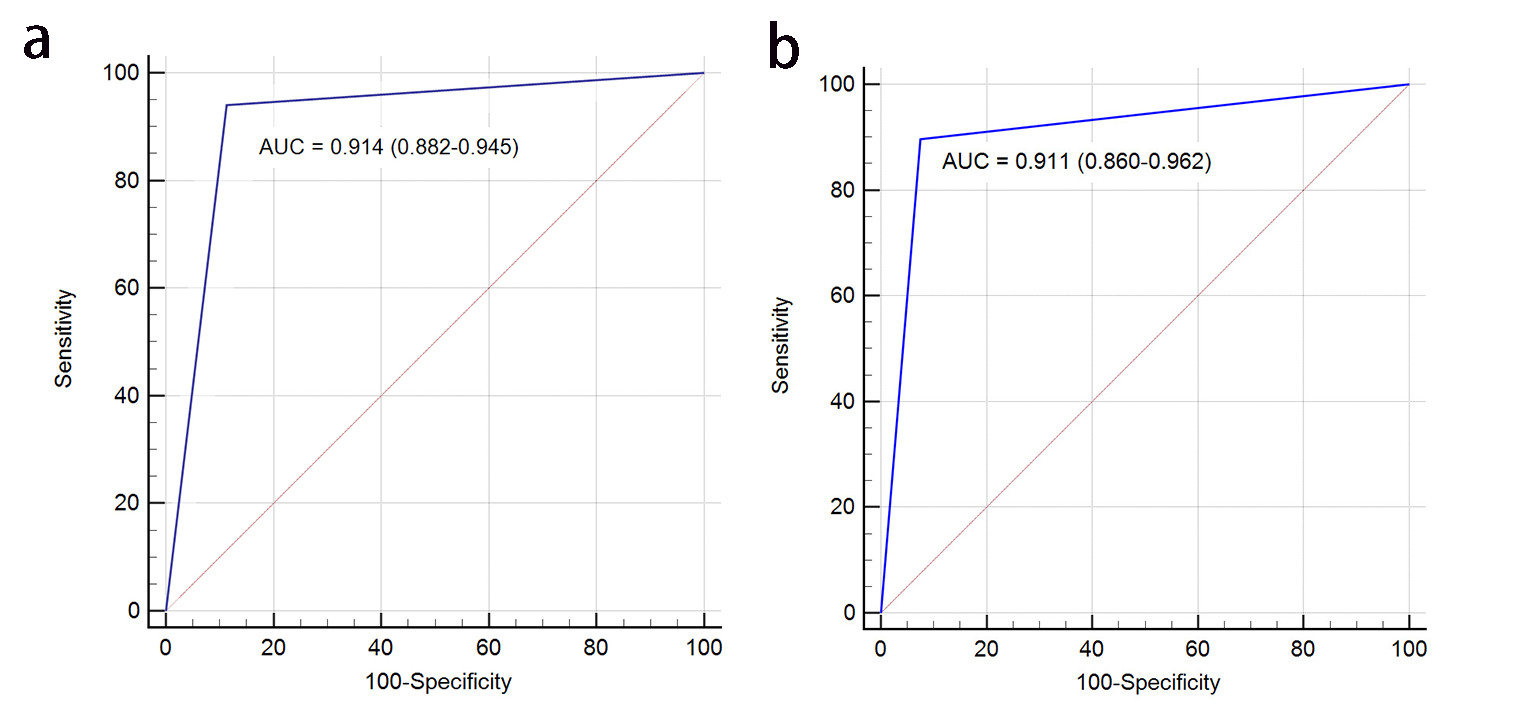
**

**Figure S1. ROC curves of the MRI-based risk scoring system in the training and validation cohorts.**

**(a)** ROC curve in the training cohort, **(b)** ROC curve in the validation cohort.
